# Healthcare providers’ and policymakers’ experiences and perspectives on barriers and facilitators to chronic disease self-management for people living with hypertension and diabetes in Cameroon

**DOI:** 10.1186/s12875-022-01892-8

**Published:** 2022-11-21

**Authors:** Amélie Mogueo, Barthelemy Kuate Defo, Jean Claude Mbanya

**Affiliations:** 1 Programme en Population, Nutrition Et Une-Santé Transnationales, Infranationales, Nationales Et Continentales (PRONUSTIC) / Program in Transnational, Subnational, National and Continental Population, Nutrition and One-Health (PRONUSTIC), University of Montreal, Montreal, (QC) H3T 1N8 Canada; 2 Department of Social and Preventive Medicine, School of Public Health, University of Montreal, 7101 Avenue du Parc, Montreal, (QC) H3N 1X9 Canada; 3 Public Health Research Center (CReSP), University of Montreal, 7101 Avenue du Parc, C.P. 6128 Succursale Centre-Ville, Montreal, (QC) H3C 3J7 Canada; 4 Department of Demography, University of Montreal, Pavillon Lionel-Groulx, C. P. 6128, Succursale Centre-Ville, Montreal, (QC) H3C 3J7 Canada; 5 Faculty of Medicine and Biomedical Sciences, University of Yaounde 1, Yaounde, Cameroon

**Keywords:** Patient empowerment, Self-management, Hypertension, Diabetes, Healthcare delivery, Healthcare providers, Policymakers, Lexicometric analysis, Thematic analysis, Sub-Saharan Africa

## Abstract

**Background:**

Hypertension and diabetes are chronic noncommunicable diseases ranked among the leading causes of morbidity and mortality in resource-limited settings. Interventions based on patient empowerment (PE) have been shown to be effective in the management of these diseases by improving a variety of important health outcomes. This study aims to examine from the healthcare providers’ and policymakers’ experiences and perspectives, the facilitators and barriers in the management of hypertension and diabetes for patient empowerment to achieve better health outcomes in the context of the healthcare system in Cameroon.

**Methods:**

We carried out a qualitative study involving three levels of embedded analysis in a public primary healthcare delivery system in Cameroon, through 22 semi-structural interviews with healthcare providers and policymakers and 36 observations of physicians’ consultations. We combined thematic and lexicometric analyses to identify robust patterns of differences and similarities in the experiences and perspectives of healthcare providers and policymakers about direct and indirect factors associated with patients’ self-management of disease.

**Results:**

We identified 89 barriers and 42 facilitators at the central, organizational, and individual levels; they were preponderant at the organizational level. Factors identified by healthcare providers mainly related to self-management of the disease at the organizational and individual levels, whereas policymakers reported factors chiefly at the central and organizational levels. Healthcare providers involved in the decision-making process for the delivery of healthcare tended to have a sense of ownership and responsibility over what they were doing to help patients develop self-management abilities to control their disease.

**Conclusion:**

While interventions focused on improving patient-level factors are essential to PE, there is a need for interventions paying more attention to organizational and political barriers to PE than so far. Interventions targeting simultaneously these multilevel factors may be more effective than single-level interventions.

**Supplementary Information:**

The online version contains supplementary material available at 10.1186/s12875-022-01892-8.

## Background

Noncommunicable diseases (NCDs) like hypertension and diabetes are long-term management diseases. Hypertension and diabetes generally coexist in the population in terms of sharing common risk factors for the development of each of these diseases [1, 2]. Hypertension is present in nearly two-thirds of patients with diabetes [1]. Their coexistence confers a 2- to fourfold increased risk of cardiovascular disease, end-stage renal disease, and death, compared with normotensive, nondiabetic adults [1]. Their pathogenic relationship is bidirectional, and their prevalence increases with age [3, 4]. The control of hypertension significantly reduces the risk of diabetic macro- and microvascular complications [5]. In resource-limited settings where inadequate or non-existence health insurance system prevails, these diseases are financially draining for patients and their families [6, 7]. The estimated prevalence of hypertension and diabetes is 32.1% [8] and 5.8% [9] respectively in Cameroon. In this country, patients’ non-adherence to therapeutic plans remains a major public health concern. This raises the need for research which can inform the design of culturally sensitive patient-centered interventions. Such interventions may sustain an integrated approach to prevent and control hypertension and diabetes, with patients’ involvement in their treatment.

The growing burden of hypertension and diabetes is a huge challenge for the Cameroonian healthcare services. People living with hypertension and diabetes require sustained engagement with the healthcare delivery system over the course of their lives [10–12]. Therefore, they need to be empowered to be able to self-manage their disease condition more than would be required for an acute health condition. Emerging as one of the general principles of the World Health Organization’s 2013–2020 global action plan for the prevention and control of NCDs [12], patient empowerment (PE) has gained increased relevance in clinical practice, policy and research [13, 14]. Recognized as a process that guides patients to be in control of all important decisions affecting their health and well-being [15], PE is a conceptual shift away from patients as ‘passive’ recipients of treatment (paternalism model) to empower individuals who are partners (partnership model) in the effective management of their health [14, 16]. PE hinges on the recognition that patient gain an experiential knowledge from living with the disease which is complementary to scientific knowledge of healthcare providers [17].

This can be facilitated at the primary healthcare which represents the level of care closest to the patient, best positioned to address the challenges of chronic disease prevention and management. However, primary care in limited-resource settings like Cameroon is traditionally geared to respond to acute episodic care needs and therefore the quality of health care for management of chronic conditions is worse, the system of care often struggle with the complexity of insufficient resources combined with inadequate access to necessary drugs and technologies [11, 18]. To strengthen the primary healthcare for better management of hypertension and diabetes, there is a need for opportunistic case finding, early detection of disease, a combination of pharmacological, psychosocial and lifestyle interventions, long-term follow-up with regular monitoring and promotion of adherence to treatment [13, 19]. Improved strategies in primary healthcare should be accompanied by public policies to prevent chronic diseases [20]. In clinical setting, we define chronic disease management as an organized, proactive, multi-component, patient-centered approach in healthcare delivery, with prepared and proactive practice team to help empower patients and families to self-manage their diseases for better health outcomes [20, 21]. Effective NCDs management interventions are vital for dealing with the rising numbers of people living and ageing with hypertension and/or diabetes. The success of these interventions depends on the overall context of political, organizational, societal, and personal factors where they take place [22]. In healthcare resource-limited settings, there is limited evidence on factors considered as barriers or facilitators to the management of NCDs, and which may contribute to patient empowerment for chronic disease self-management. The views of healthcare providers and policymakers who are generally key actors in the implementation of NCDs management interventions may help identify potential challenges or obstacles at different levels of the healthcare system for optimizing such interventions. Some factors identified from different key stakeholders’ groups [23–26] tend to vary from one context to another or from one stakeholder group to another. Henceforth, the knowledge base remains limited in resource-limited settings like Cameroon regarding healthcare providers’ and policymakers’ standpoints on potential barriers or facilitators in the management of NCDs for patient empowerment to achieve better health outcomes. The aim of this study is therefore to contribute to fill this gap for the NCDs self-management for people living with hypertension and diabetes in Cameroon.

### Conceptual framework

**Fig. 1:**
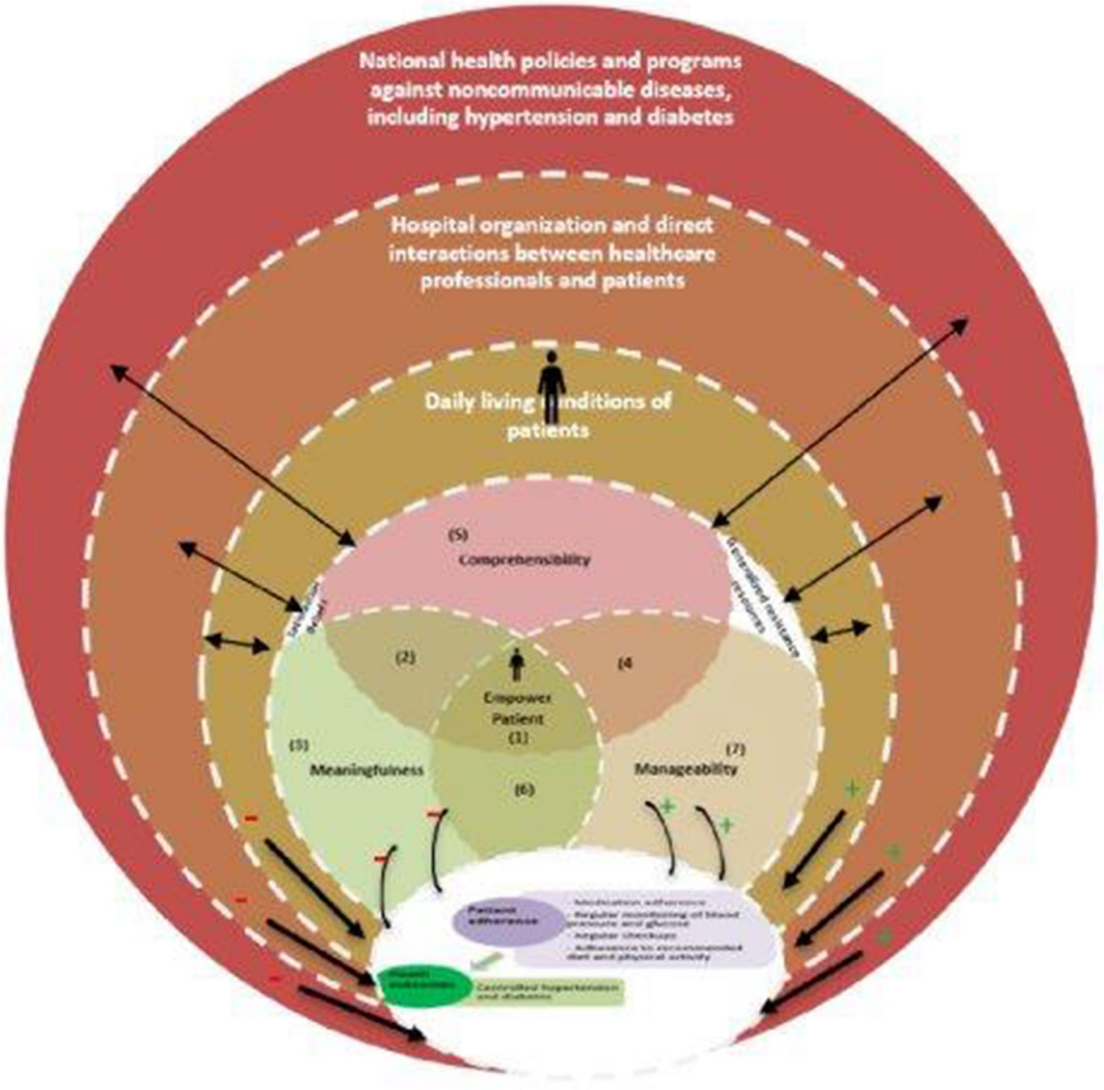
An integrated framework for understanding barriers and facilitators to chronic disease self-management for people living with hypertension and diabetes

Different conceptual models exist in literature, but the chronic care model (CCM) developed in the 1990s by Wagner et al. [20] is a dominant framework, which is effective in guiding the delivery of health care services for chronic conditions. To fit different contexts, the WHO adapted this model to integrated chronic care conditions (ICCC) [20]. However, this CCM or ICCC may not be directly applicable to limited-resource settings. So, to adapt this model of care to the specific context and constraints of limited-resource settings, Beaglehole et al. [21] propose ‘best fit’ framework synthesis (BFFS), which is also an adaptation of the CCM developed based on diabetes and hypertension. This model is very close to the primary care model for NCDs propose by Kane et al. [18] for Sub-Saharan African countries. To reflect the realities of our study context, we reframed the existing models to create a new framework which includes components at the macro (policy), meso (healthcare organization), and micro (patient and family) levels (Fig. 1). We recognize that positive policy environment that supports care for chronic conditions is essential to empower patients for better health outcomes, this includes legislation, leadership, policy integration, partnerships, financing, and allocation of human resources identified in ICCC framework [20]. The healthcare organization with the interactions between patients and health care providers is the operational level which focuses on screening, prevention and control of the diseases as describe by Kane et al. [18]. This includes cases finding, modified risk factors, standard diagnosis and treatment, referral pathway, adherence and follow up, tasks shifting, training of staff, decentralized care, essential diagnostics and medicines, systematic monitoring and evaluation [18], which indirectly influence patient empowerment in the self-management of NCDs for better health outcomes. The micro level represents the daily living conditions of patients surrounded by families and surroundings, where the patient empowerment happens for better health outcomes [22]. At this level, PE process is influenced by several direct factors including the level of education, profession, marital status, presence of support, motivation, and attitude of patients [22, 27].

PE approach has been influenced by several theories of health behavior change [28–31], including the salutogenic theory proposed by Antonovsky [32–34]. We consider an integrated approach linking the salutogenic theory [32] to the health belief model including patient belief [31] and to the patient satisfaction theory [35]. Indeed, PE in the management of hypertension and diabetes is shaped by a multiplicity of interacting factors from health policies/programs, hospital organization and the direct interactions between the healthcare providers and everyday living conditions of patients as present above. To better understand from the healthcare professionals and policymakers’ views and experiences which of these factors are facilitators or barriers in the management of NCDs to the development of patients’ abilities to self-manage their disease for better health outcomes, we constructed our approach from the salutogenic theory [32]. This theory operationalizes PE approach in two main concepts: sense of coherence (SOC) embodied by comprehensibility, manageability, and meaningfulness, and generalized resistance resources (GRR). This theory suggests that to be empowered and have better health outcomes, patients need to develop a strong SOC (comprehensibility, manageability, meaningfulness) with available GRR (internal and external resources) [34, 36]. SOC refers to a patients' capability to use generalized resistance resources (internal and external resources) to understand factors affecting their health (comprehensibility), to be able to better manage the diseases (manageability) and to looking at life as worth living (meaningfulness) [36]. A stronger SOC is predictive of a production of good health, while the presence of GRR, internal and external resources are prerequisites to develop a stronger SOC [34]. The central dimension of SOC is the “meaningfulness”, which refers to the motivational component and constitute the driving force of life [36]. For patients, when the situation makes sense, they become more inclined to seek, to identify and use resources to strengthen the other two components (comprehensibility, manageability) and to regain good health. In other words, with a great meaningfulness, regardless of the level of comprehensibility and manageability, the patient seeks to move up (Fig. 1).

Our approach also recognized that PE hinges not only on patients’ SOC and available GRR, but also on patients’ belief [31] and patients’ satisfaction [35], as both influence all three dimensions of SOC, and the patients’ ability to use the existing GRR. Indeed, patients’ beliefs influence their perceptions and experiences of their disease. Personal beliefs influence the understanding of the disease (comprehensibility), daily actions taken to manage the disease (manageability), and motivations behind each choice (meaningfulness). Patients’ satisfaction as a positive evaluation of healthcare delivery services means that the more patients are satisfied, the more they will adhere to treatment and hence develop the ability to self-manage their disease and vice-versa (manageability). Patients’ satisfaction also influences their motivation to self-manage their diseases and to make sense of the process (meaningfulness). Therefore, patients’ beliefs and satisfaction influence the SOC (comprehensibility, manageability, meaningfulness), and its integration with existing resources (GRR) used by patients. Improving the different components of health behavioral change can then improve patients’ adherence to treatment and ultimately improve their health outcomes. Improved health outcomes can in turn be a source of motivation for people to maintain change, which may reduce the utilization of healthcare services.

## Methods

### Study aim, design and setting

We aimed to investigate, from healthcare providers’ and policymakers’ experiences and perspectives, which factors from health policies/programs, hospital/organization, direct interaction of patients with the health system and their everyday living conditions, are barriers or facilitators to the development of PE.

We carried out a qualitative study involving three levels of embedded analysis within the healthcare delivery system of Cameroon to investigate PE in managing hypertension and/or diabetes [10]. The first level is the patients and their family in everyday living conditions. The second level consists of health districts implementing primary healthcare programs developed at the third/central level which is responsible for developing policies and strategies, coordination and regulation.

The study setting was a public primary healthcare district hospital (PHCDH), where the healthcare delivery system is operationalized. This PHCDH is the first reference for 11 public and private health centres, the patient’s gateway to the healthcare system. This PHCDH has a department of internal medicine where over 30 patients daily suffering from hypertension/diabetes were cared for by a team of healthcare providers: six nurses, two general practitioners (GP), and four specialist physicians (SP).

### Participants

Using purposeful sampling and snowball technique, we selected key participants made up of 12 healthcare providers (six nurses, two GP, and four SP), two hospital policymakers (HPM) (also called hospital administrators) and eight national policymakers (NPM) who met our inclusion criteria. The eligibility criteria included working directly with patients suffering from hypertensive and/or diabetes (for healthcare providers) or being directly involved in hospital organization or health policies/programs related to hypertension or diabetes (for HPM and NPM). We recruited the healthcare providers and HPM at the hospital, and the NPM from the ministry of public health.

### Data collection

The study was approved by the Health Research Ethics Committee of the University of Montreal, Canada, and the Cameroon National Ethics Committee. The interviewer obtained informed consent by a written signature from participants prior to each interview.

Prior to data collection, we piloted and refined data collection instruments based on three 40-min interviews with health providers (two GP and one SP), they were not part of the actual study. From 07 January to 29 March 2019, we collected data through semi-structured interviews (*n* = 22), observations of consultation by the SP for outpatients (*n* = 29) and by the GP for inpatients (*n* = 7), and documents related to the management of hypertension and diabetes (*n* = 9). Fieldwork notes were taken. Each interview was conducted at the participants’ workplace, starting with the healthcare providers, then the HPM and the NPM. We had access to the information needed to get in touch with participants through the head of the department of internal medicine. The first author conducted all interviews for 30–60 min using interview guides (Additional file 1) and deliberated with the second author when significant decisions had to be made. The first author transcribed interviews. The second author independently read all transcripts with corresponding audiotapes and found no discrepancies. To ensure data confidentiality and participants’ anonymity, we replaced all participants’ names with alphabets and numbers; once completed, all audiotapes were destroyed.

Direct and passive observations for 10 to 30 min, of consultations of patients took place in SP offices and patients’ hospitalization rooms using observation guides (Additional file 2). Documents reviewed included health education (*n* = 3), healthcare services for patients (*n* = 2), and national policies/programs on hypertension/diabetes management (*n* = 4).

### Data analysis

We did a qualitative analysis by combining different complementary techniques.

First, the documentary data were closely read to understand the national action plan against chronic NCD, to have details about the patients and the delivery of health service at the PHCDH. Then, all transcripts and field notes were entered into the software package QDA Miner for coding [37]. Interview data were thematically analyzed [38] by the first two authors. The analysis was conducted by combining deductive and inductive approaches. We undertook an inductive content analysis approach using constructivist thinking, allowing themes to emerge [38]. To check for consistency, we did triangulation by seeking different data sources in the study and by crosschecking different points of view of participants [39]. Findings from different sources and methods used led to concordant findings. We focused on aggregating codes into key themes and assigning them to two categories: facilitators and barriers; organized according to comprehensibility, manageability, meaningfulness, internal and external resources, beliefs, satisfaction, adherence and patients’ health outcomes.

We used the IRaMuTeQ software to perform lexicometric analysis in order to explore differentiated discourses on self-management of chronic disease for patient empowerment to achieve better health outcomes [40]. The textual corpus was prepared based on our conceptual framework. The textual corpus preparation is a fundamental step in the lexicometric analysis: after merging all textual content to be analyzed, a thorough text verification was undertaken to ensure that there were no spelling errors, that acronyms were standardized, that synonym use was standardized, and that the entire textual corpus was accurate for analysis. IRAMUTEQ allows the identification of textual classes (e.g., sub-codes in Additional file 3) based on the textual corpus which in turn result in definitive categories (e.g., themes in Additional file 3). The lexical analysis using IRAMUTEQ streamlines data processing when there is a large textual volume and increases methodological rigor by retrieving information with statistical bases to analyze the meaning of terms/words in the corpus of analysis. This process allows for a thorough qualitative data analysis, providing transparency regarding the process of inference and analysis of the processed textual corpus, thereby increasing the possibilities of deepening analysis and reliability of results. More specifically, we did a word cloud analysis and similarity analysis [41]. The word cloud analysis displays the lexicon of words associated with the corpus in the form of a graph where the size of the words is proportional to their frequency. The most quoted words are placed in the center. The similarity analysis allows a co-occurrence analysis presented in the form of graphs of associated words. The words are the vertices of the graph, and the links represent the co-occurrences between them.

## Results

Sociodemographic characteristics of participants.

The study enrolled 22 participants: 12 healthcare providers (six nurses, two GP, and four SP) and 10 policymakers (two HPM and eight NPM) (Table 1). Half of participants were women, but the national policy makers were only men. Nurses and GP were younger while SP and policymakers were older. The majority of participants were married. The nurse’s education level was lowest compared to physicians and NPM who were the most educated. The number of years working on patients with hypertension or diabetes was lower for nurses, compared to physicians and policymakers who had more years of experience with hypertension and/or diabetes management.

**Table 1:** Sociodemographic characteristics of participants

Participants / characteristics		Nurses	General physician	Specialist Physician	Hospital policy makers	National policy makers	Total
Sex	Female, n	5	2	2	1		10
	Male, n	1	/	2	1	8	12
Age	< 40 (30–40), n	4	2	1	1	/	8
	≥ 40 (40–58), n	2	/	3	1	8	14
Education level	Bachelor's degree, n	6	/	/	/	/	6
	Master's degree, n	/	/	/	1	/	1
	Doctorate in medicine, n	/	2	/	/	/	2
	Doctorate + Specialization, n	/	/	4	1	8	13
Number of years of providing care to patients with hypertension and/or diabetes	< 10 (2–9), n	6	2	2	1	1	11
	≥ 10 (10–29), n	/	/	2	1	7	11
Marital status	Married, n	5	/	3	2	8	18
	Single, n	1	2	1	/	/	4

### Healthcare providers’ and policymakers’ experiences and perspectives

Following the multilevel healthcare system in Cameroon, we present findings from health care professionals and policy makers’ perspective and experiences on barriers and facilitators linked to PE in managing hypertension/diabetes grouped following our framework and substantiated by illustrative quotes from participants.

We identified 89 barriers (Additional file 3) and 42 facilitators (Additional file 4) at central, organizational, and individual levels; both barriers and facilitators were more represented at the organizational level.

### Comprehensibility

**Fig. 2:**
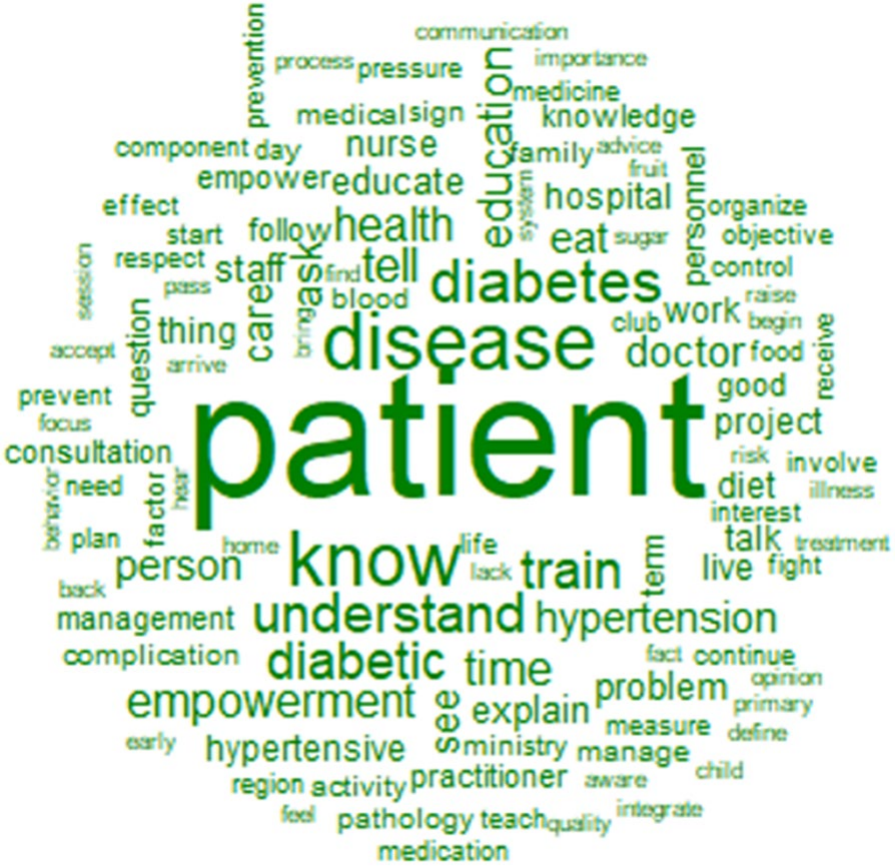
Words used to describe the comprehensibility with their frequencies correlating to their size

Figure 2 shows the words used to describe comprehensibility, with their frequencies correlated to their size. The top 12 words used to describe comprehensibility were patient, disease, know, diabetes, understand, train, diabetic, tell, education, time, health, and empowerment (Fig. 2).

**Fig. 3:**
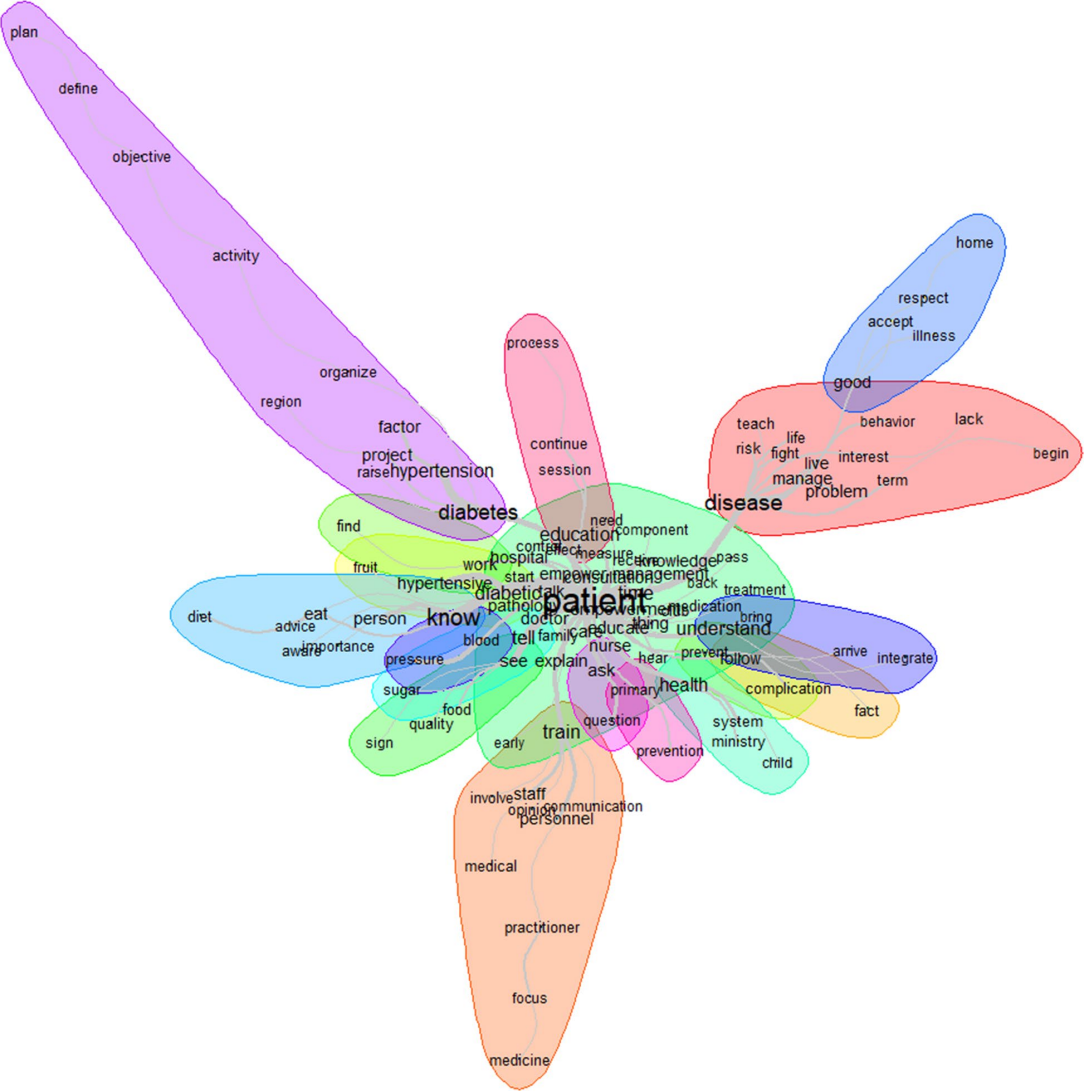
Similarities and links between the different words used to describe the comprehensibility

The similarity analysis showed that the link between patient, disease, and understanding of the disease through words like disease, diabetes, train, know, and understand, was highly represented (Fig. 3). That link was stronger when talking about the problem (disease, diabetes) than the solution (train, know, understand, education).

Patients, nurses, and general physicians did not participate and did not know much about national hypertension and diabetic policies or programs, but some specialist physicians had participated in the development of these health policies. Although the national program against chronic NCD did not include “patient education” in hospital, awareness campaigns for the general population were generally organized. However, the strategy was not always adapted to reach as many people as possible. Specialist physicians in the hospital did the training of nurses on diabetes and hypertension. In the hospital, not all staff were interested in the continuum training as shared:

“Well, there are training for some staff only, I can't say I'm one of those people. For example, my colleague also did not receive the training. Because if there is continuous training, the fact that they do not involve all the staff is a real problem.” (Nurse, female).

Therefore, most nurses and the general physicians did not have enough knowledge about the management of the diseases and the concept of “patient empowerment”. Because of the patient education counselling (PEC) club in PHCDH, some nurses were trained beforehand on the knowledge and management of hypertension and diabetes to educate and follow-up patients every week to support them in managing their disease. Nevertheless, these trained nurses were sometimes moved to other departments and replaced by new untrained nurses, as described by one hospital policymaker:

“I'm going to surprise you but sometimes there are some nurses in the community who do not know how to do it, how to manage diabetes because they have not been trained. Those who are trained for this reason are sometimes sent to other departments.” (Hospital policymaker, female).

In addition, some physicians did not always take the time to explain the disease to patients and involve them or their caregivers in the management of the disease. All specialist physicians were aware of the meaning of “patient empowerment”, from their research or given the recommendation from the government. Some were able to speak many local languages in addition to French and English, which helped facilitate the communication with patients and their caregivers. For example, one specialist physician recounted:

“For those who speak French and English it OK, and I can say that for me I have no problem, I am polyglot so, I speak several languages, sometimes I use the vernacular languages to communicate the message. I can also use the patient's language for their educational needs.” (Specialist physician, female).

At the patient level, the main barrier was low levels of education, which make it difficult to understand the physician’s recommendations to be able to manage the disease. The educated patients were the ones who were apt to explain their needs during consultations, thereby allowing physicians to prescribe a therapeutic plan adapted to their situation.

“… there are certain things that the patient cannot understand if he does not have a certain level of education. If he is not educated it will be very difficult, the educated patient is easy to empower. But we work in areas where patients are mostly non-educated.” (Specialist physician, female).

### Manageability

**Fig. 4:**
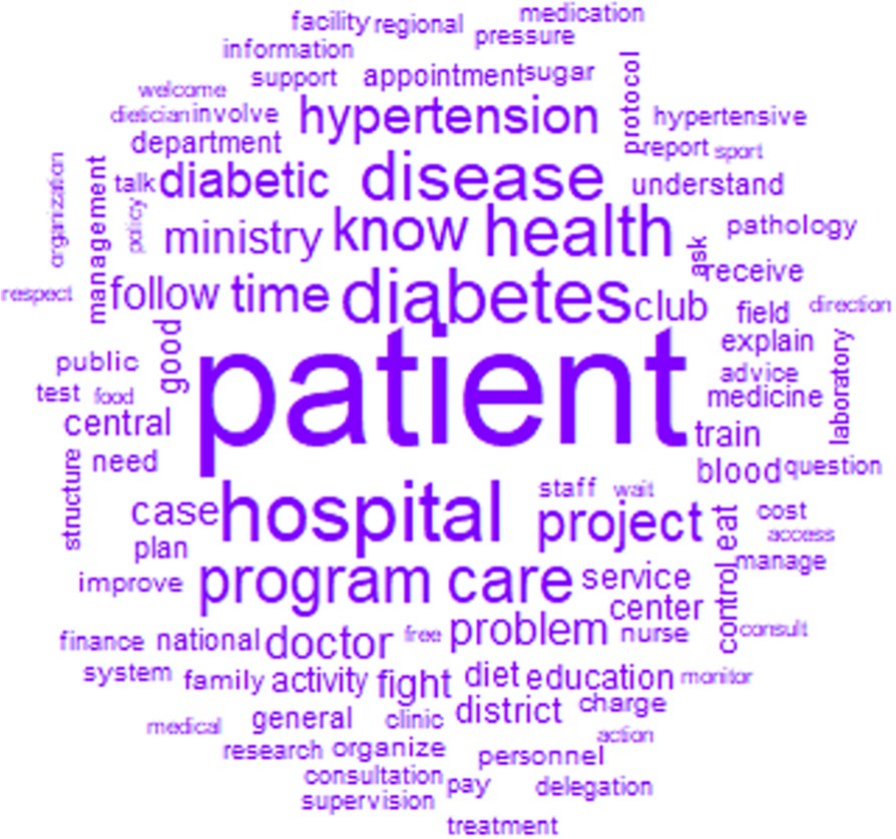
Words used to describe the manageability with their frequencies correlating to their size

The words used to describe manageability with their frequencies correlated to their size are shown in Fig. 4. The top 12 words used to describe manageability were patient, hospital, diabetes, health, disease, program, care, know, project, time, hypertension, and diabetic (Fig. 4).

**Fig. 5:**
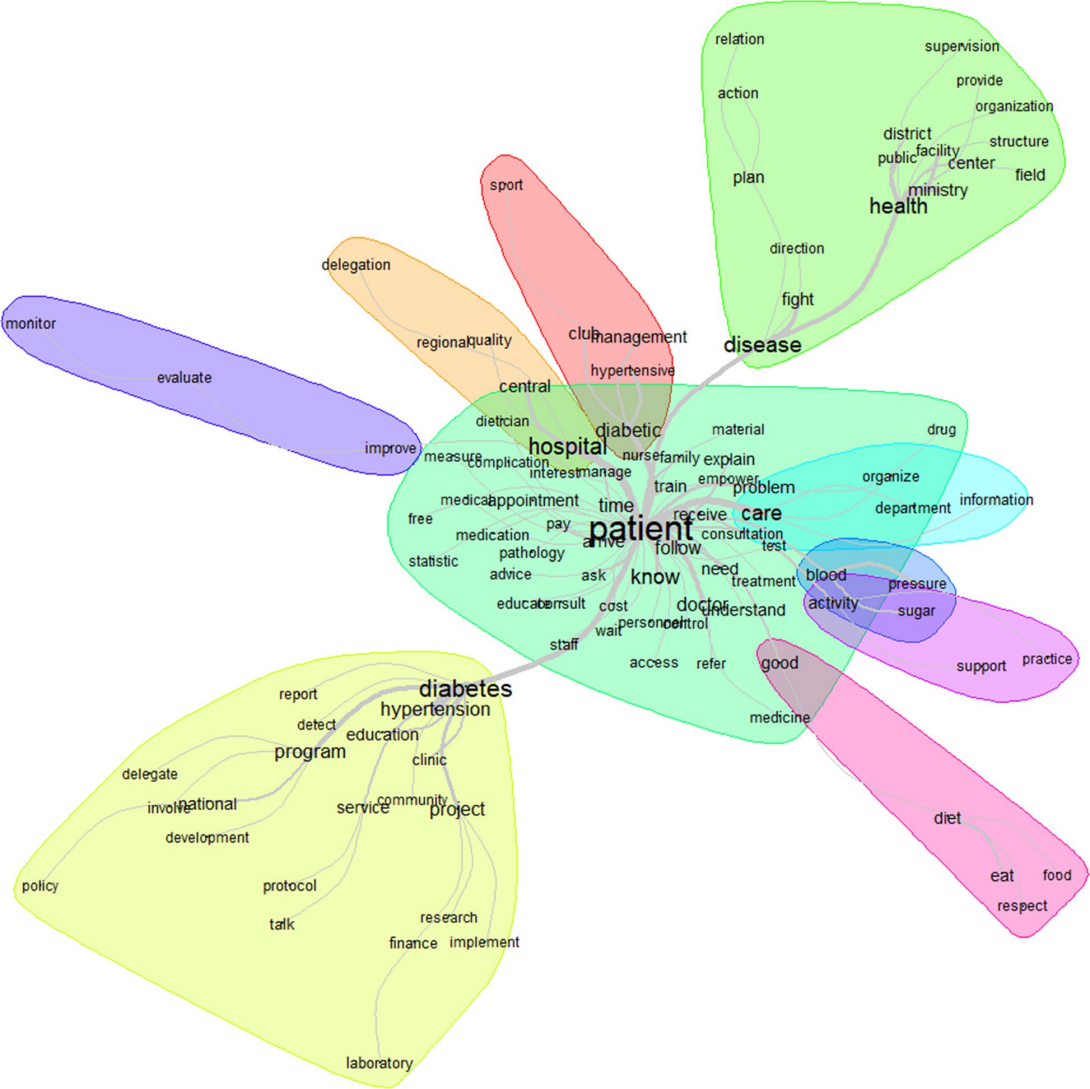
Similarities and links between the different words used to describe the manageability

The similarity analysis showed that the link between patient, disease, and health care delivery through words like diabetes, disease, diabetic, hospital, care, and activity, was highly represented (Fig. 5). That link was stronger when talking about the problem (diabetes, disease, diabetic) than the solution (hospital, care, health).

The health system was very centralized with a non-operational national plan against chronic NCD. The health system still focused on infectious diseases, and there was little deployment for the control of chronic NCD such as diabetes and hypertension. For example, there were few or no health promotion or disease prevention initiatives, even though this was a key strategy in the fight against chronic NCD. Moreover, the health information system was almost non-existent. This lack of data made it difficult to monitor the hospital's activities. There was no accountability and no system to control the health services delivery and products, including the distribution of drugs. Similarly, there was no protocol for physicians to guide their decisions and actions, or to standardize the healthcare service delivery for all patients in the hospital. The only existing care protocol in the hospital was for nurses developed by the ministry of health, as a national policymaker indicated:

“We developed protocols for the management of hypertension and diabetes, … I had the privilege of leading the development of protocols and so, we wrote protocols for diabetes, hypertension and other chronic diseases at the district hospital level for nurses.” (National policymaker, male).

Although there were several partnerships with non-governmental organizations (NGO) with expertise in the management of hypertension and diabetes, the centralization of the health system made it difficult for them to implement and follow-up their program. Additionally, there was no strategy from the ministry of health to sustain and expand NGO projects implemented in the PHCDH. Thus, NGO faced difficulties in agreeing with the chief medical officer of the hospital, whose decisions and actions were sometimes contrary to the program's objectives regarding hypertension and diabetes, as one of the national policymakers pointed out:

“… for example, we trained doctors or nurses to take care of diabetic patients at the internal care department and a few weeks later we find the same nurse is assigned to the maternity department, because it is the chief who decides the turnover of his staff and we can't do anything about it.” (National policymaker, male).

The PHCDH was still focusing more on delivery services adapted to infectious diseases, with the absence of the unit for health promotion or prevention of the disease. Pharmaceutical companies, who exerted indirect pressure on them through their representatives, generally influenced the physicians’ prescriptions. This was possible because there were no national guidelines for the prescription of medicines and delivery of care for hypertension and diabetes; the guidelines generally used by physicians were developed by international or foreign institutions, and were not always well suited to the PHCDH setting as one of the specialist physicians stressed:

“Yes, it is more about my personal experience with patients because I spent a lot of time with them. The guidelines I use are in relation with the recommendations of international organizations, but in general, they do not include the social situation of these patients.” (Specialist physician, male).

The centralization and the management of healthcare delivery affected the quality of the health services through the access to the health care services, the waiting time to see the physician and their relationship with patients, and the time permitted to each patient. The presence of PEC club and the task shifting strategy adopted by some physicians helped to decongest the hospital. However, this also created an overload of work for some nurses who found themselves with various tasks to do and who were not always in providing care in good working conditions.

“In general, we do the "task shifting", we empower patients but also staff. Of course, those who come do not have that spirit, but in a short time they get involved in everything we do, we explain to them the merits of everything we are doing and the logic. … so, in everything we do we try to involve them.” (Specialist physician, male).

At the individual level, the difficulty in changing lifestyle or living alone or going from one hospital to another or from traditional medicine to physician's recommendations, negatively affected the follow-up of these patients and their health outcomes, as reported by one of the nurses:

“…The patient who move from one hospital to another, or from a traditional medical system to here is a problem, he moves from one health center to another, so, we do not follow him up continuously, it difficult for him to change and sometimes his health declines faster.” (Nurse, female).

### Meaningfulness

**Fig. 6:**
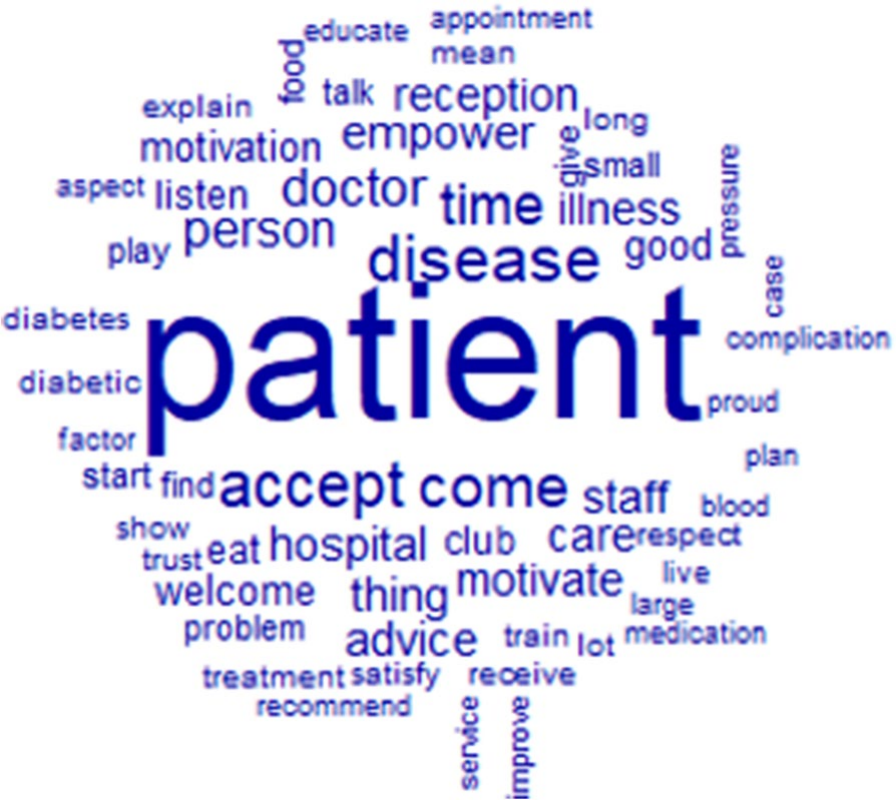
Words used to describe the meaningfulness with their frequencies correlating to their size

The words used to describe meaningfulness with their frequencies correlated to their size are shown in Fig. 6. Patient was the most represented word. The top 12 words used to describe meaningfulness were patient, disease, accept, come, time, doctor, hospital, empower, advice, staff, reception, and motivate (Fig. 6).

**Fig. 7:**
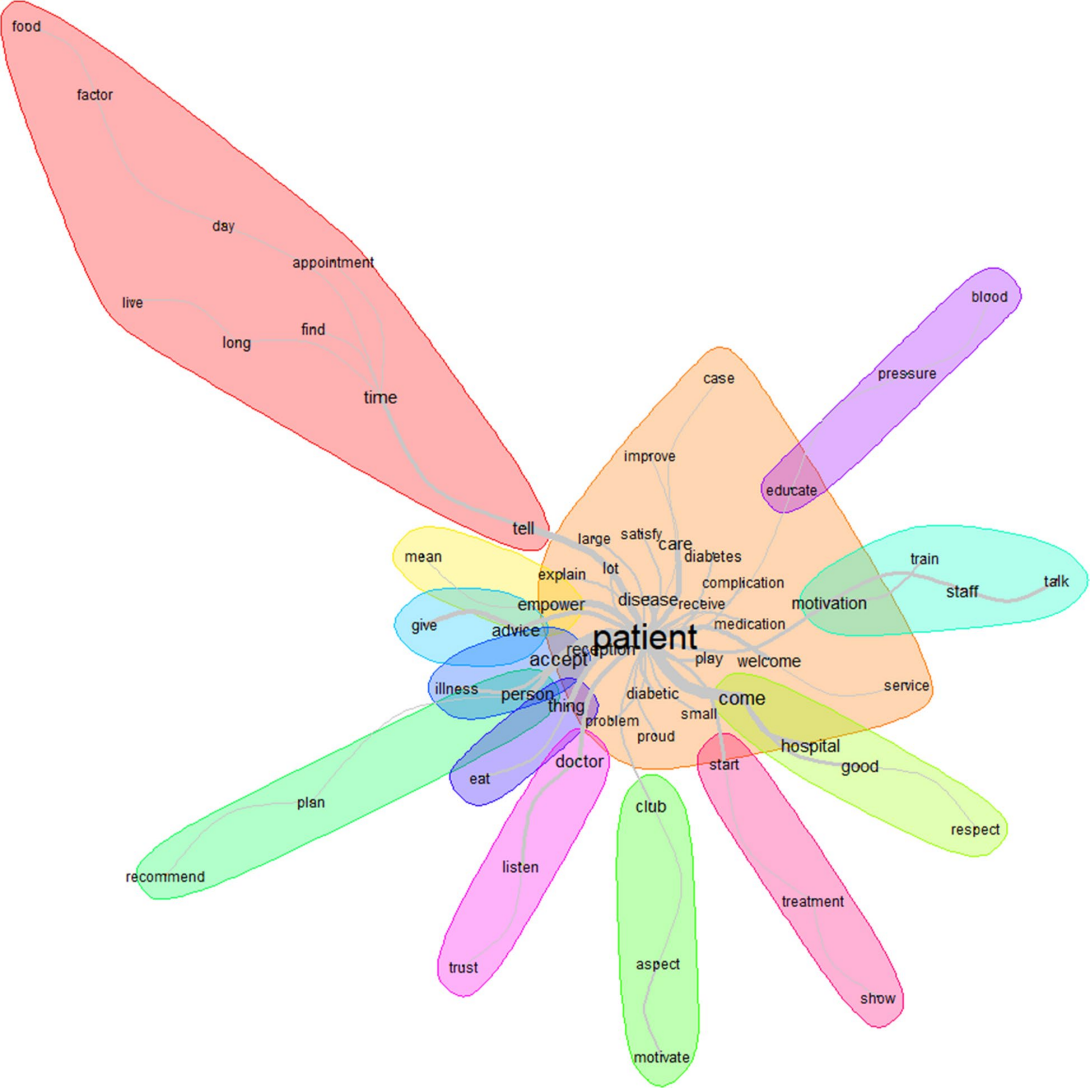
Similarities and links between the different words used to describe the meaningfulness

The similarity analysis showed that the link between patient, and tell, accept, come, motivation, doctor, and advice, was highly represented (Fig. 7); they were mainly positive words.

The principal barrier at the central level was a matter of priorities, as the government was less engaged to invest in the management of chronic NCD. This led to the lack of engagement of the staff for continuous training that included self-management strategies and PE approach to health service delivery. This made them feel helpless in how to motivate their patients. A relationship of trust between some physicians and patients was a real facilitator to boost patients’ and their caregivers’ motivation.

“They listen to them because that is the right thing to do. When you listen carefully to your patient, you will gain his trust, and this trust we have it here between our doctors and patients.” (Hospital policymaker, Male).

The fear of the disease and not trusting the healthcare providers’ competencies negatively affected patients’ motivation. Nevertheless, accepting the disease or the will to live longer positively influenced patients’ motivation to adhere to their treatment and recover their health as described:

“When the patient accepts the disease, that the disease is a chronic disease, he participates because you can tell him what to do. When he accepts that now I must eat this and not this, I must walk, that means yes, he begins to be empowered, and follows his treatment.” (Specialist physician, female).

### Resources

**Fig. 8:**
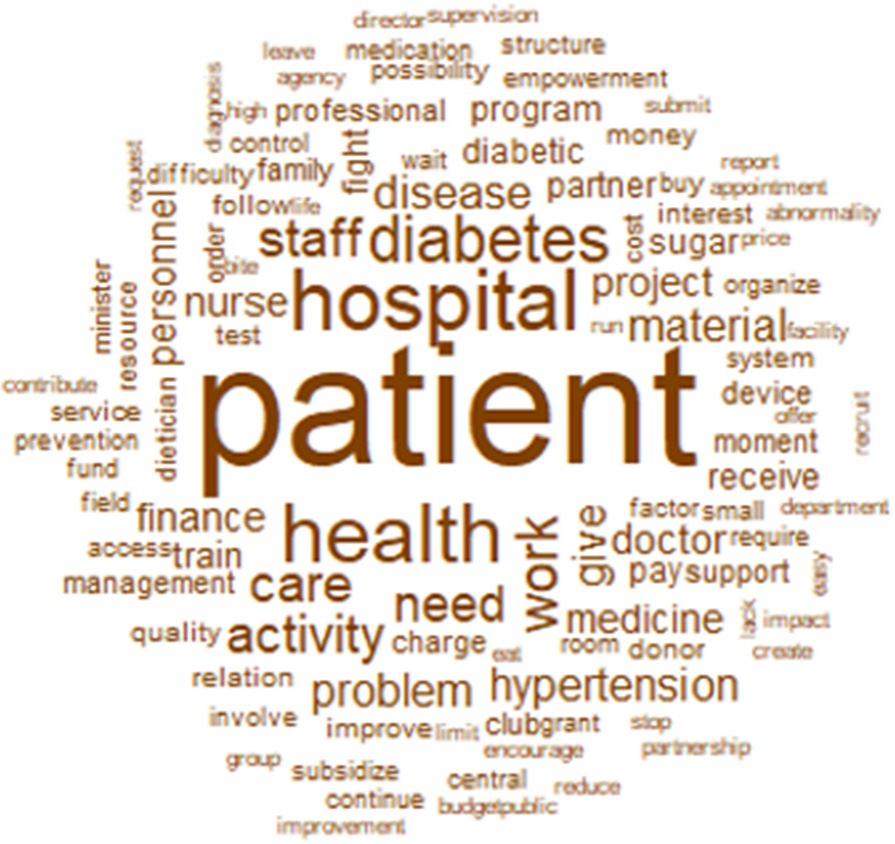
Words used to describe the resources with their frequencies correlating to their size

Figure 8 displays the words used to describe resources with their frequencies correlated to their size. The top 12 words used to describe manageability were patient, health, hospital, diabetes, work, care, staff, activity, need, disease, problem, and give (Fig. 8).

**Fig. 9:**
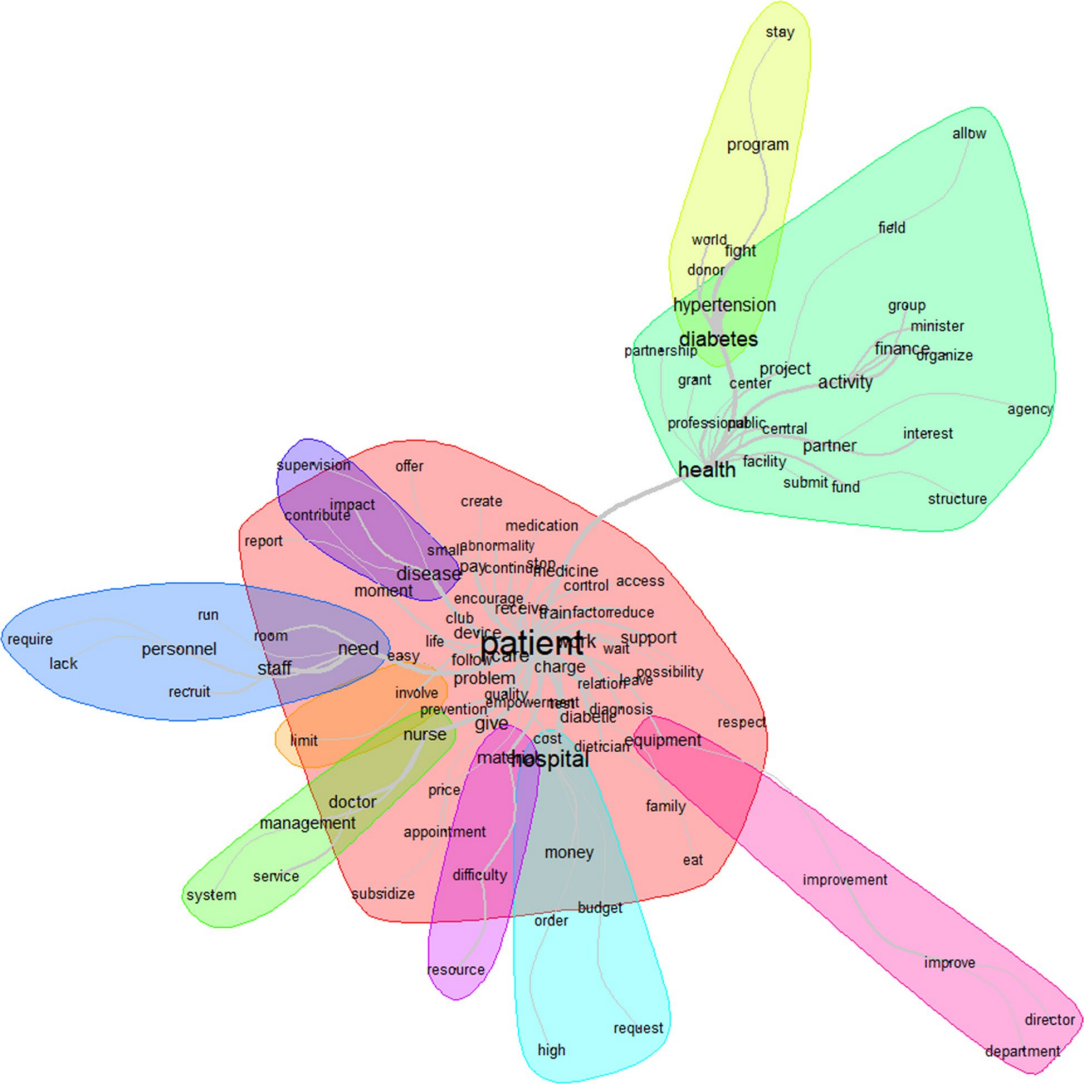
Similarities and links between the different words used to describe the resources

Patient was the most represented word. The similarity analysis showed that the link between patient, and health, diabetes, hospital, need, disease, and material, was highly represented (Fig. 9). That link was almost the same when talking about the problem (diabetes, need, disease) and the solution (health, hospital, material).

The Cameroonian health system is characterized by the absence of a universal health insurance system, and patients and their caregivers usually pay themselves the costs of healthcare services and products. This was an important barrier for many of them who did not have enough money to defray those costs. In addition, the budget allocated to the management of chronic NCD was very small compared with that for infectious diseases, there were not enough human resources involved in the management of chronic NCD and no subsidy for products or services for patients suffering from hypertension or diabetes. It was only through partnerships with some NGO that patients and their caregivers had access to some subsidized health products and services.

“… very often, the financial envelope allocated to us is not the one we asked for, because there are budgetary constraints, and the ministry is funding more infectious diseases. They do not make the resources available for the management of diabetes and hypertension. So, we are just trying to adjust with what we have. We must tell the truth, the ministry of health does not currently make anything available to us for the fight against diabetes and hypertension, when we say follow-up of the patient, you see that you ask patients to go buy drugs and equipment; many cannot buy because they do not have money. So, it is the NGO who funds all activities here.” (Hospital policymaker, male).

The barriers identified at the organizational level included the lack of space in the PHCDH to manage all patients with hypertension or diabetes who were usually received in a corridor, no room for PEC club meetings and the lack of human resources (nurses and dietician). The funds were very limited and did not allow for the recruitment of additional staff which the hospital needed. The salaries of healthcare providers—mainly nurses—were very low and there was a lack of resources for continuing training. Moreover, the working conditions were very difficult for some nurses. Some of the available products or equipment in the hospital were defective, many were not subsidized and were very expensive for the patients and caregivers, and some were frequently out of stock as reported by one of the nurses:

“… they (NGO) made available many products for the club, and when it finished, we go directly to order more. But lately they were out of stock, the patients of the club were angry, it hurt me because they did not deliver on time, the person who had to come with did not come.” (Nurse, female).

The presence of many specialist physicians at PHCDH positively influenced the process of patient empowerment, as they had expertise and experience with patients having hypertension and diabetes. They shared this knowledge and expertise between the staff through continuous training and tasks shifting. They also helped the poorest patients and caregivers who could not afford their medications by giving them the ones they received from laboratories or donations, as reported by one of the physicians:

“… We help them, especially patients who cannot afford products, we give them appropriate treatment and sometimes we help them with the drugs, we give them the drugs for free to really help them in their care management.” (Specialist physician, male).

At the patient level, the identified barriers were the lack of finance to purchase recommended medicines or healthy foods, the lack of secure places for exercising or walking, and the lack of help or family support or being solely responsible for one’s disease. In contrast, patients who were financially able to afford the costs of their care, had their own equipment to measure blood glucose or blood pressure, were married or had children tended to be facilitating factors of the process of patient empowerment. One healthcare provider shared her experience:

“We also found for some patients that, we could not use them when talking about empowerment, we had to go through the family. For example, wives do it very well for their husbands, sometimes it is the children who pay and manage everything around the patient.” (Nurse, female).

### Beliefs

We found that the acceptance of the disease and the estimation of susceptibility to the consequences in addition to the severity of the disease needed to be well-thought-out for patients living with hypertension and/or diabetes. As patients' beliefs influence their perceptions and experiences with the disease [31], they influence the understanding of the disease (comprehensibility), the daily actions taken to manage the disease (manageability), and the motivations behind each action taken (meaningfulness). So, believing that spiritual forces caused the disease, that alternative medicine was the solution, or that it was an incurable disease, was an important barrier for patients to follow their recommended therapeutic plan.

“… It is her husband, they say we are going to treat her with alternative medicine, but we can see she is not controlled at all, and there are already complications.” (Nurse, female).

### Satisfaction

We found that patient satisfaction depended on prior expectations of care. Ten constructs have been identified in literature to be considered for patient satisfaction: accessibility, availability of resources, continuity of care, efficiency/outcomes of care, finances, humanity, information collection, information provision, welcoming environment, quality/competence [35]. Three of these constructs were identified as causes of patients’ complaints or barriers to PE including the waiting time, the organization of the hospital, and the attitude of some nurses. In contrast, two of them were mentioned facilitator to PE. It is the quality of the healthcare services from the reception to follow-up related to the dynamism and the good energy of the PEC club had a positive impact on patient satisfaction.

“The follow-up at the hospital helps them because we organized a diabetic and hypertensive club for these people, it's every Thursday, they come to the appointment, I explain things to them and you see it changes the atmosphere, there are a lot of them who like the good vibes…” (Nurse, Female).

**Discussion**

From the perspectives and experiences of healthcare providers, hospital policymakers and national policymakers, we identified interacting factors pertaining to the three levels of the healthcare system that influence the development of PE in the management of hypertension and diabetes in Cameroon. Barriers and facilitators to PE identified were similar within participant groups and different between groups. Policymakers tended to be aware of factors at the central level, while healthcare providers reported mainly barriers and facilitators at the organizational and patient levels.

### Comprehensibility

Patients who had access to health information were those who had either a television, a radio, or an internet access. This group were poorly represented in our study in a resource-limiting setting like Cameroon where in 2014; only 10% of the general population had access to the internet [11]. Hence, although access to suitable information could have been a facilitator in the process of PE, its low level significantly reduced its potential benefits for patients. Indeed, the interest of the group screening and awareness campaigns lies in their ability to empower a larger number of people at once. But to be effective in the medium to long term, group campaigns should have the adapted communication channels, be continuous with the follow-up of those at risk or sick [12, 42]. Such prevention strategies for hypertension and diabetes were absent from the national action plan against chronic NCD in Cameroon [11]. Even when there were some proposed strategies in the national action plan that could favor PE, their implementation at the subsequent levels of the health care system was ineffective. The World Health Organization’s global action plan 2013–2020 against NCD recommends "patient empowerment" as a core principle to guide all interventions [12]. Yet, the 2016 national action plan against chronic NCD in Cameroon, developed after the World Health Organization’s recommendations (2013), does not include these recommendations and therefore, does not allow the healthcare system and patients to take advantage of the expected benefits. While there is, to our knowledge, no prior directly comparable research with which to relate these findings, several barriers to the development process of PE that emerged from our study were also been identified elsewhere. For instance, the funding constraints and hierarchical bureaucratic structure of the Cameroonian healthcare system, characterized by a high degree of centralization and complex administrative procedures, remain a dominant feature of the state of health systems in Africa [43].

At the hospital level, nurses and general physicians were not aware of the national plan against chronic NCD, which also reflected the fact that they were not involved in the development of such plan. This plan did not integrate the continuing education policy for health professionals. Specialist physicians occasionally participated in continuing education when international opportunities arose. In the hospital, they were responsible for providing continuing education to nurses. However, due to the lack of time and organization of care, nurses seldom received such education in a timely and coordinated fashion. Thus, the nursing team had a heightened lack of knowledge about the management of hypertension and diabetes, although they spent most of the time with patients. This was reflected in their lack of competence and sense of powerlessness to help patients and a lack of trust in them by patients. Most specialist physicians, although trained and knowledgeable, devoted little time to patient education during consultations. Various systematic reviews found time constraints to be one of the most frequent barriers of shared decision-making [19, 44]. The more time spent on patient education involving them in the decision-making process, the more likely they are to be empowered to make healthy choices for better health outcomes [36]. It is not just about giving patients' time, it is also about patients being able to explain their health problem and being able to understand the physician's recommendations to potentially apply them [19, 23]. Educated patients were those who expressed their health needs better and understood such recommendations.

### Manageability

The organization of the healthcare system, from planning to implementation of interventions plays a key role in how patients are able to use existing resources to take action and develop their autonomy [27]. However, while strategic planning at the central level revealed that consideration of chronic NCD increased in recent years, it still remained low compared to infectious diseases [10]. The excessive centralization including leadership and governance of the health system in Cameroon made it difficult to advance the health goals forward by underperforming, regardless of the resources it was able to master [33]. There is a need to more balanced/horizontal approach; focus on prevention, education, and awareness of the disease’s management, involving all the actors of the healthcare system [45].

Although some healthcare providers were informed and often consulted, they were not involved in the decision-making process at the central level and, as a result, the action plan adopted at the national level does not always meet the local needs of the hospital. For example, there was a lack of nurses in the internal medicine department of the hospital compared to the number of specialist physicians, which affected the working conditions characterized by an overload of work for some nurses and consequently a poor quality of healthcare. As a process, PE is built continuously through interactions with health professionals [46]. Patients were not informed or involved in the decision-making process, at either the policymaking or care management level. Palumbo et al. [47] recommend involving patients in both strategic and organizational decisions aimed at patient empowerment.

The lack of a well-developed health information system did not allow a good assessment of the magnitude of the situation of hypertension and diabetes in PHCDH. If availability of a robust data collection system and analysis for decision-making is one mark of a good health system [43], PHCDH was operating at a snail pace to improve the health conditions of its people. Data collection and utilization were likewise poor; although structures for data collection, reporting and feedback existed, their level of functionality was suboptimal. Data recordkeeping and reporting was still largely paper-based. What is needed is the strengthening of the capacity of the PHCDH through skills training, improving staffing, and regularly supporting supervision [18]. Therefore, increasing funding with strict accountability for such health centers and improving activities for tackling NCD are not an option but a priority in Cameroon and similar African countries [18].

For patients, there is need to not only raise patients’ awareness about the benefits of engagement but also to encourage and support patients’ increasing responsibility and leadership in disease management [22]. For healthcare providers, there is need for formal training or regular reflection on self-management approaches, so it may not become tools to control or to blame patients [19, 24], but it can help increase clinicians’ confidence in self-management approaches skills. Making small changes, such as talking less and listening more, were found helpful for clinicians [36].

### Meaningfulness

Since the government did not make chronic NCD a priority, this was reflected in the delivery of healthcare by the volume of resources allocated and a low level of commitment of healthcare providers who were less motivated to help patients to self-manage their disease. The less motivated the staff is to engage in patient self-management, the less likely patients will be able to control their own disease [48]. To better assist and motivate patients to become effective partners in their care, training, resources, and tools are needed. A relationship of trust built between some physicians and patients based on ethical values of the medical profession where the patient is the center of health care delivery [27], was a real facilitator to boost patient and family motivation. In addition, involving healthcare staff in the development of hypertension and diabetes programs can stimulate and increase their motivation for involving patients in their healthcare [47].

At the patient level, the fear of disease, the unwillingness to change, and not trusting the healthcare providers’ competencies also affected negatively patients’ motivation to follow the therapeutic plan. Accepting the disease or the will to live longer positively impacted patients’ motivation to adhere to the therapeutic plan for recovering health. This highlights the importance of patient education to understand the disease and related symptoms, to make a correspondence between their actions and the positive effects on the development of state of health [36], so they can accept the situation and know they can still live longer by following the recommended therapeutic plan. For patients depressed by the loss of a family member, offering psychologist services to help them regain control of the disease could be a good option, as recommend by the World Health Organization in the management of chronic NCDs [12].

### Resources

GRR identified as a barrier to PE included financial (e.g., low budget), material (e.g., defective work products/equipment), immaterial (e.g., not enough time), intellectual (e.g., lack of appropriated skills), humans (e.g., shortage of nurses), structural (e.g., space in the hospital), and personal resources (e.g., lack of help/support). These factors directly or indirectly influence the ability of patients to act and actively engage in the management of the disease [49]. For example, the inadequate or nonexistent health assurance system and the awfully low budget allocated for the management of chronic NCD in the healthcare system in Cameroon [10] given the 15% of the national budget recommended in Abudja in 2001 [43] makes patients and caregivers bearing treatment costs [6]. As a low- and middle-income country, people were not able to pay, which greatly reduces adherence to the treatment plan [6]. Indeed, patients who are unable to pay medical or hospital bills tend to be unable to follow their treatment plan, which typically includes hospital visits, laboratory tests, medications, follow-ups, and lifestyle changes. In other words, the inability to pay medical or hospital bills characterizes the lack of external resources that patients could use to better manage their diseases. The government needs to make available access to essential medicines and services for patients.

The use of partnerships with some NGO helped to subsidize certain products and services, to facilitate access to healthcare and follow-up for patients. This also created the availability of work equipment and a good technical platform which improved the quality of healthcare services. Indeed, studies have shown that the quality of healthcare and feeling supported in managing the disease strongly influence patient’s engagement and involvement in the decision-making process and the motivation to be adherent to the therapeutic plan [44, 50].

### Belief

Our findings suggest that believing that spiritual forces cause the disease, or that alternative medicine is the solution or that it is an incurable disease did not allow patients to change their lifestyle which is one of the most important dimensions in the occurrence and development of the disease. This raises the importance of continuing education and counselling for patients and the general population about the causes and development of chronic NCD [36]. To change patients’ belief that negatively affect their health, there is need of long-term strategies, because just as it takes time to learn and build a belief about the disease [51], it will take as much or more time to unlearn before the new knowledge/belief about the disease is adopted [52].

### Satisfaction

Patient satisfaction is the patient's positive evaluation of healthcare dimensions including structure, process, and outcomes [53]. Studies have shown that, the more patients are satisfied the more they will be engaged in the management process of the disease, what increase the adherence to the therapeutic plan with more chance of recovery [42]. Considering patient satisfaction as an indicator of the quality of services, cost efficiency, and population health, improving dimensions they did not appreciate (e.g., waiting time) while sustaining dimensions they appreciated (e.g., experience with PEC club) will contribute to achieving and sustaining productive PE in chronic NCD management [42].

### Strengths and limitations

A major strength of this study is the innovative use in combination of thematic and lexicometric analyses of qualitative study data. This allowed uncovering similarities and differences across the different levels of the healthcare system in a resource-limited setting like Cameroon, in the experiences and opinions of healthcare providers and policymakers regarding the development of patients’ empowerment in the management of NCD such as hypertension and diabetes. This helped increase the validity of our findings. Another strength is that healthcare providers and policymakers have different experiences that inform their opinions on what happens in the entire healthcare system, which lend support to considering healthcare providers’ and policymakers’ recommendations about structural, process, and systemic changes which may promote successful PE and related outcomes.

Some limitations should be mentioned. The number of participants may appear small, and this study was limited to one of the main hospital. However, qualitative research is meant to capture detailed information from few, representative participants. Their views may reflect the Cameroonian healthcare setting or similar resource-limited settings, and may not be transferrable to other settings. Moreover, there is always a consideration of authenticity when observers know they are being observed; unfortunately, there was no way to avoid this influence on actions.

## Conclusions

Our multilevel analysis of PE in the management of hypertension/diabetes found that health providers-identified factors mainly related to self-management of the disease at the hospital/organizational and individual levels, whereas policy makers-identified factors chiefly focused at central and hospital/organizational levels. Barriers were more than twice as many as facilitators; both were less identified at the central and individual levels compared to organizational level of health care. The identified factors at different levels suggests the need to pay more attention to them in the multilevel patient empowerment interventions in limited-resource settings like Cameroon. Even if interventions focused on changing patient factors, such as knowledge or motivation, there is a need for more interventions addressing organizational and political barriers to PE. Although highly motivated patients may become empowered, without clear opportunities and invitations, many patients will not. The preponderance of hospital/organizational-level factors over national/central and individual levels suggests they should receive more attention in the multilevel PE interventions in limited-resource settings.

## Supplementary Information

**Additional file 1.** Interview guides for policymakers and healthcare providers.

**Additional file 2.** Observation’s guide of interactions between health professionals, patients, and their families during consultations.

**Additional file 3.** Barriers to patient empowerment in the management of diabetes/hypertension.

**Additional file 4.** Facilitators to patient empowerment in the management of their diabetes/hypertension.

## Data Availability

All data generated or analysed during this study are included in this published article [and its supplementary information files].

## Abbreviations

GP: General practitioner
GRR: Generalized resistance resources
HPM: Hospital administrators/policymakers
HTN: Hypertension
NCD: Noncommunicable diseases
NGO: Non-governmental organizations
NPM: National policymakers
PE: Patient empowerment
PEC: Patient education counselling
PHCDH: Primary healthcare district hospital
QDA: Qualitative Data Analysis
SOC: Sense of coherence
SP: Specialist physician
T2D: Type 2 diabetes
