# Supplementary material for: Healthcare providers’ and policymakers’ experiences and perspectives on barriers and facilitators to chronic disease self-management for people living with hypertension and diabetes in Cameroon
Source: BMC Prim Care. 2022 Nov 21;23:291. doi: 10.1186/s12875-022-01892-8 (PMC9680136; doi:10.1186/s12875-022-01892-8)
Supplement: Supplementary file 1 — Additional file 1. Interview guides for policymakers and healthcare providers. [file 12875_2022_1892_MOESM1_ESM.pdf]

Additional file 1. Interview guides for policymakers and healthcare providers

Interview guide for policymakers (central and organizational levels)

Identification:

- Date ………………; Time: beginning ………., end ……………

- Name and surname:

- Age: ………. Sex: ……….

- Marital status:

- Education level:

- Occupation:

-Number of years working on hypertension and diabetes:

1) How would you describe your involvement in policies/programs for the control of chronic non-communicable (NCDs) diseases including diabetes and hypertension in Cameroon / your hospital?

- Program preparation, implementation, monitoring and evaluation.

2) What do you know about the patient empowerment approach?

Resources, intelligibility, manageability, meaningfulness, sense of coherence, adherence, etc.

Pause for clarification if need ……………

3) How does the national plan against chronic NCDs, mainly hypertension and diabetes, enable or hinder patients to be empowered and self-managed the diseases, to be adherent to their treatment plan and have the expected health outcomes?

- Planning: strategies, objectives, allocation of resources (material, financial, human, infrastructure, etc.), subsidization of products and services, etc.

4) How does the implementation of the national chronic NCDs program, mainly hypertension and diabetes, in the hospital enable or hinder patients to be empowered and self-managed the diseases, to be adherent to their treatment plan and have the expected health outcomes?

- Availability, accessibility, utilization of resources, quality of healthcare and services, patient education, etc.

5) How does direct healthcare and services from health professionals enable or hinder hypertensive and/or diabetic patients to be empowered and self-managed the diseases, to be adherent to their treatment plan and have the expected health outcomes?

- Availability, acceptability, number of health care professionals, nature of relationships between health care professionals and patients, etc.

6) What factors do or do not promote the empowerment of hypertensive and/or diabetic patients to self-managed the diseases, to be adherence to the treatment plan and have better health outcomes at the individual, family, community level?

Interview guide for healthcare providers

Identification:

- Date ………………; Time: beginning ………., end ……………

- Name and surname:

- Age: ………. Sex: ……….

- Marital status:

- Education level:

- Occupation:

-Number of years working with hypertensive and/or diabetic patients:

1) How would you describe your involvement in the management of chronic non-communicable (NCDs) diseases including diabetes and hypertension in the hospital?

2) What do you know about the patient empowerment approach?

Resources, intelligibility, manageability, meaningfulness, sense of coherence, adherence, etc.

Pause for clarification if need ……………

3) How does the national plan against chronic NCDs, mainly hypertension and diabetes, enable or hinder patients to be empowered and self-managed the diseases, to be adherent to their treatment plan and have the expected health outcomes?

- Planning: strategies, objectives, allocation of resources (material, financial, human, infrastructure, etc.), subsidization of products and services, etc.

4) How does the organization of healthcare and services in the hospital enable or hinder hypertensive and/or diabetic patients to be empowered and self-managed the diseases, to be adherent to their treatment plan and have the expected health outcomes?

- Availability, accessibility, utilization of resources, quality of healthcare and services, patient education, etc.

5) How does direct healthcare and services from health professionals enable or hinder hypertensive and/or diabetic patients to be empowered and self-managed the diseases, to be adherent to their treatment plan and have the expected health outcomes?

- Availability, acceptability, listening, nature of relationships with patients, etc.

6) What factors do or do not promote the empowerment of hypertensive and/or diabetic patients to self-managed the diseases, to be adherence to the treatment plan and have better health outcomes at the individual, family, community level?
