# Supplementary material for: Healthcare providers’ and policymakers’ experiences and perspectives on barriers and facilitators to chronic disease self-management for people living with hypertension and diabetes in Cameroon
Source: BMC Prim Care. 2022 Nov 21;23:291. doi: 10.1186/s12875-022-01892-8 (PMC9680136; doi:10.1186/s12875-022-01892-8)
Supplement: Supplementary file 2 — Additional file 2. Observation’s guide of interactions between health professionals, patients, and their families during consultations. [file 12875_2022_1892_MOESM2_ESM.pdf]

Additional file 2. Observation's guide of interactions between health professionals, patients, and their families during consultations

Activity	Health professionals	Patients	Families (if present)	Notes
Physical setting				
Duration of visit				
Topics of discussion				
Individual activities				
Nature and quality of interactions				
Participation (active vs passive)				
