# Supplementary material for: Healthcare providers’ and policymakers’ experiences and perspectives on barriers and facilitators to chronic disease self-management for people living with hypertension and diabetes in Cameroon
Source: BMC Prim Care. 2022 Nov 21;23:291. doi: 10.1186/s12875-022-01892-8 (PMC9680136; doi:10.1186/s12875-022-01892-8)
Supplement: Supplementary file 3 — Additional file 3. Barriers to patient empowerment in the management of diabetes/hypertension. [file 12875_2022_1892_MOESM3_ESM.pdf]

Additional file 3. Barriers to patient empowerment in the management of diabetes/hypertension

Health system	Mega codes (8)	Sous codes (18)	Themes (89)	Representative quotes
**Central level (25)**	SOC (21)	Intelligibility (3)	1- No involvement of health professionals / patients in the elaboration of national action plan against chronic NCDs	1- *"I have no idea, I'm just someone who works here for the hospital, I do not know how they do that. It is for the ministry of health to do that. I do not know, no idea. ... we are there at the operational level ... so I tell you that the operational level is the level of application, we simply apply what is decided at the top."*
			2- No program for diabetics and hypertensive patients' education	2- *"Education for the prevention of hypertension and diabetes, this project does not do it. It is something we could have done if we had a minimum of resources, we could design microprograms for the education of the population in the local language, because all the villages have community radio with programs that speak in the local language so..."*
			3- Lack of knowledge of national action plan against chronic NCDs (Diabetes/HBP) by health professionals	3- *"... to my knowledge, I do not know ... no no no, the question this is not at my level. Excuse me to tell you that, I'm here only to work with the patients, I give what I can give to help, I have no idea on things like that ... sincerely about to the diabetes hypertension program I do not know."*
		Manageability (15)	1- National action program against chronic NCDs (Diabetes/HBP) is not operational	1- *"The program so it exists on paper, but the implementation is not yet effective. It requires funding. When giving objectives to a program it must also give the budget to achieve these goals - so maybe they are still at this stage there."*
			2- system is more focused on communicable diseases	2- *Health system is much more oriented towards infectious diseases such as malaria, tuberculosis, HIV/AIDS, and vaccination. But for chronic diseases, there is still very little data... if I want to give my personal thought, I think Cameroon would benefit from setting up a diabetes hypertension program like the malaria or HIV program."*
			3- Not enough awareness / screening campaign adapted to reach as many people as possible	3- *"... as a health system, the government should develop more health campaigns or conferences or other large-scale training, to be able to educate patients. And at the level of health professional, each staff should take time whether the patient is diabetic or hypertensive to give them a therapeutic education. There is not enough awareness, we must continue to raise awareness about the importance of management, diagnostic, living with the disease, knowing the associated factors and the importance of prevention"*
			4- No effective action plan for health promotion and prevention of diabetes and HBP	4- *"I think if we have to act ... I think that in our country we have a major problem on health promotion because that is the main aspect of combating chronic diseases, we have to put a lot of emphasis on promoting health, low-salt diet, reduce fat intakes, the promotion of physical activity this kind of thing."*
			5- Difficult to create partnership to finance the diabetes and HBP projects	5- *"... so overall, we really have a lot of difficulty to access the partner, because very often you have to know and have contact with people interested. ... but as I said there is not many partners interested with chronic NCDs... The advocacy is not strong enough because of few field data..."*

			6- Only one person for all the supervision at the central level	6- *"I'm here at the regional public health delegation as a focal point for diabetes and hypertension, i.e. I am the one who coordinates related activities in all district hospitals ... and all hospitals in the region. So, these activities range from staff training to the equipment of health centers, monitoring the quality of care, collection of statistics, monitoring of community screenings, and return information in terms of challenge occurrence, complications, incidences of complications related to diabetes/HBP. So, all of this is what I have to do daily."*
			7- Poor or no develop health information system: use of social media like "WhatsApp" to manage data	7- *"Every end of the month, our technical worker send data by whatsApp and when possible, they send the physical copy of the report. ... it was not known exactly how many diabetic patients we had in the health facilities. ... the current flaw is that the data recovery is not that effective, quality of the data still a concern to the system ... we see that there is a lot of lost and can be duplicates so, the image is not clear and sharp."*
			8- No monitoring of the Hospitals, PHCDH	8- *"... the department of public health is not available for us to check our activities against diabetes and hypertension... they should check every week to evaluate our actions and not wait to say at the end that things are wrong, or it is well done, we need to work together."*
			9- No health system insurance	9- *"You should know that health policies are developed in a particular context and the context of Cameroon at the moment is a bit special because, as I said there is no universal health insurance, patients still pay out of pocket."*
			10- Centralization of the health system, impossible for researchers to act without the Ministry of Health	10- *"Basically, the NGO cannot go on the ground without me, because it is in collaboration with the ministry of public health and the delegation represents the ministry of public health. So, if he goes without me, they will not open the doors to him. So, in general he needs us to go on the ground, because all the hospitals in which the project is implemented are public hospitals ... therefore it needs the agreement of the regional delegation to go into these structures, otherwise they might not receive him. The NGO has no authority to come and check if the recommended prices are being met, or by putting supervisors on site."*
			11- No strategy to sustain and expand NGO projects in hospitals	11- *"We still have them (NGO) until June 2020. The ministry of health should take over but very often, operationally it is not done, either the ministry leaves it to the hospitals to say continue, and if for the director of the hospital diabetes and hypertension are not very important, it neglects, and we close the unit and life resumes as before."*
			12- Low/uncontrol of drug supply and distribution channel	12- *"... you know that in Cameroon, generic drugs are not yet used as drugs that must be used by doctors. So, doctors are free to prescribe even specialized drugs that are expensive. ... drugs remain a big problem because even when we talk about generic the problem of drug quality remains. We do not have the ability to check the quality of certain drugs which means that some drugs can come from India, from China or anywhere without us having the opportunity to check if the active ingredient is sufficient in the drugs we give to our patients. ...other problem that we have is the parallel networks of fake drugs in place."*

			13- Poorly paid of healthcare professionals	*13- "The salary here is very low, to the point people are forced to look for complementary resources to survive. That is what created corrupted doctors, they ask to the patient to pay them directly. It is true it is not normal, but you must know that the cost of living is very high, and that the salary is low. So, you must in a program like that, provide for the motivational elements. If the salary were high the problem would not arise, we had to ask the staff to do their job or we put them out, when the salary is very low you cannot hold a very rigorous speech."*
			14- No guideline/protocol for health professionals in hospitals	*14- "The central level has developed protocols but are not yet disseminated, and doctors have never issued a directive that requires them to operate according to these protocols, this is the stage that has yet to be put in place."*
			15- No direct contact with patients	*15- "... we do not have direct contact with patients, it is the reports we receive from health facilities that make us believe that things are going well or not. …systematically we have no contact with the patient. ... I am telling you currently the ministry does not have the opportunity to follow up or assess quality of care or patient empowerment verification.*
		Meaningfulness (3)	1- It is a matter of priorities	*1- "... so depending on the level of priority gave at that time - the ministry of finance will give the financial budget at the hospital which is limited because health it is not a priority for the government. So, you must choose what you are going to prioritize over the others.*
			2- IT is matter of burden of chronic NCDs, not as high as the burden of infectious diseases	*2- "A nurse in a health center sees many more cases of infectious diseases in the context of our health centers here, the nurse is overwhelmed by infectious diseases like diseases of children, diarrhea, bronchitis, malnutrition, pregnant women problem. From time to time, you can have a hypertensive or diabetic patient, but it is not common, which means that asking a nurse like that to devote his time to a diabetic patient is a challenge."*
			3- Government is less motivated to invest in the management of chronic NCDs	*3- "... even the government do not yet perceive very well the magnitude of the problem, because if you do not have data, you cannot convince anyone. So, if the problem is not perceived by the government, i.e., the advocacy is not strong enough"*
	RRG (4)	Internal resources	/	/
		External resources (4)	1- Low budget allocated to the management of chronic NCDs (diabetes and HBP)	*1- "... very often, the financial envelope allocated to us is not the one we asked for, because there are budgetary constraints, and the ministry is funding more infectious diseases. They do not make the resources available for the management of diabetes and hypertension. So, we are just trying to adjust with what we have. We must tell the truth, the ministry of health does not currently make anything available to us for the fight against diabetes and hypertension, when we say follow-up of the patient, you see that you ask patients to go buy drugs and equipment, many cannot buy because they do not have money. So, it is the NGO who funds all activities here."*

			2- No budget for health promotion and prevention of chronic NCDs (diabetes and HBP)	*2- "The Ministry of Health does not make the resources available for prevention against diabetes and HBP. So, if I want to go to Biyem-Assi to supervise a diabetes and HBP screening campaign, I will see my supervisor to tell him I have to go to Biyem-Assi, you must give me a car or money to pay the transport. He will tell me that there is nothing on the ministry's budget lines, nothing is planned for diabetes and hypertension."*
			3- Not enough human resources involved in the management of chronic NCDs at the central level	*3- "At the central level, I must admit that in terms of staff we do not have enough. In my department, there are 5 with an office in charge of supervision, an office responsible for the healthcare, and an office responsible for prevention. So, overall, there is a lack of people, and it requires a lot of time, resources, and staff so, this level does not work well."*
			4- No subsidy of products / services for diabetic and hypertensive patients	*4- The ministry can help these patients to have products because, when we say follow-up of the patient, you see that you ask patients to go buy drugs and equipment, many cannot buy because they do not have money. So, for e.g., the ministry of health can subsidize the blood pressure monitors for patients to have a good blood pressure monitor."*
	Beliefs		/	/
	Satisfaction		/	/
	Adherence and health outcomes		/	/
**Organizational level (39)**	SOC (20)	Intelligibility (5)	1- Not qualified healthcare professional for diabetics / hypertensive patients	*1- "I'm going to surprise you but sometimes there are some nurses in the community who do not know how to do it, how to manage diabetes because they have not been trained. Those who are trained for this reason are sometimes sent to other departments."*
			2- Health professionals do not provide therapeutic education to diabetic/hypertensive patients	*2- "Yes, we have to do some work on it because we have realized that the patients in Cameroon and this is often the case in Africa are not involved in the management of their affection, sometimes people do not even know why they are being hospitalize for. ... there are patients who come to the hospital, no one has ever explained to them what they are suffering from, we do not educate them."*
			3- Not all healthcare staff are involved in the continuing training	*3- "Well, there are training for some staff only, I cannot say that I am inside those people. For example, my colleague also did not receive the training. Because if there is continuous training, the fact that they do not involve all the staff is a real problem."*
			4- No strong scientific evidence that clearly describes the situation with chronic NCDs (Diabetes/HBP)	*4- "... what I'm going to say will not be scientific because I do not have evidence to say that patients are empower. But for us, we want to measure this, the attendance, complications that occur in patients, and in the management of the patient, do they know to prevent the occurrence of hypoglycemia of coma etc. But there is no data to say that."*
			5- Lack of Knowledge of health professional about "patient empowerment"	*5- "Patient empowerment, this word no I have not heard yet, we have not had any teaching or presentation on it... I think empowering we do it every day, but the word empowerment is what I had in mind, also I think it is a communication right?*

				It is a perpetual exchange between patient and doctor, so that he can be really impregnated in their treatment."
		Manageability (12)	1- Difficulties working with chief medical officers of the hospital, uncooperative	1- *"... for example, we trained doctors or nurses to take care of diabetic patients at the internal care department and a few weeks later we find the same nurse is assigned to the maternity department, because it is the chief who decides the turnover of his staff and we cannot do anything about it."*
			2- Not data base at the PHCDH in the management of patients and follow up	2- *"... we do not know exactly how many diabetic patients we had ... we did not formally assess the impact of the program because I told you at the beginning, we started from scratch, nothing was done or documented at the hospital of Biyem-assi ... The statistics are not exhaustive, we are talking about number and frequency, we must increase the number of variables we use. ... like complications, what drug he took, how much medication he took, the cost of his treatment ..."*
			3- No patient records or a patient data collection system for good follow-up	3- *"The problem is data collection which is a bit administrative and does not really fit into doctors and nurses training. For e.g., when you have seen a patient, you must keep the data and each patient must have an identification number that he can use everywhere. So, when you ask a nurse after consultation to fill patient file correctly, it is complicated and difficult. We have an archiving service but that is cramped. ... we consult with notebooks the patient returns with his notebook and the notebook is not well maintained.*
			4- High concentration of patients at PHCDH	4- *"... we have a high concentration of patients at the Biyem-Assi hospital, because the other eighteen are not supported by donors so, we have a double-speed in Biyem-Assi."*
			5- Problem with accessibility to healthcare and products (strategic, geophysics, technical or financial accessibility)	5- *"... in fact my understanding of empowerment is that, when I see empowerment, I see the possibility that the patient can access care to be organize and follow doctor recommendations. This requires a good technical tray, and very often they complain about the inaccessibility of the doctor and they must wait for hours. ... and access to medicines, glucometers, etc."*
			6- No standardized protocol for the management of diabetic and hypertensive patients	6- *"Yes, it is more about my personal experience with patients because I spent a lot of time with them. The guidelines I use are in relation with the recommendations of international organizations, but in general, they do not include the social situation of these patients. So, there are more personal tricks that I try to share with other practitioners, and I think with that, there will be success especially in African environment. So, there is not really a guideline in relation to that."*
			7- Doctors' works are influenced by of pharmaceutical companies	7- *"In our country doctors are very influenced by pharmaceutical companies, protocols are not yet mandatory for prescribers. Most of the time, medicines are provided by various laboratories; Americans, Europeans, Indians, and the doctors are under pressure from the delegates who are the representatives of these laboratories, because each laboratory would like the doctors to prescribe its drug."*

			8- Communicable diseases are favored over NCDs	*8- "Medical staff are much more focused on infectious diseases, like malaria, tuberculosis, HIV and vaccination, but for chronic NCDs there are still very few people involved."*
			9- No room for diabetes club meetings	*9- "Certainly we have our diabetes club, but we do not really have a room where you can say that this for the club. If only we can find a place all participants in the club. We are scattered, sometimes the club meets in the hallway, the privacy is not there, there are some people who come for something else, but finally following the discussion of the club."*
			10- Centralization of management of healthcare at PHCDH	*10- "I say this because everything is centralized, the patient who arrives for a blood glucose check is forced to line up at the cash register to pay, initially it was not the case but for anti-corruption, we centralized everything. So, the waiting time is long for the patient. … I should come, receive my patients, and leave. So, whoever went to line up, he is at risk to come when I am no longer there. So, it is annoying …"*
			11- Heavy workload and difficult working conditions for some health professionals	*11- "We have to increase the number of people in the staff because you see that there too much to do. So, we still must recruit nurses to assist others. He would have to have more nurses because we really do not have enough in this hospital, sometimes it is a bit difficult, there are already four doctors I think it is enough but compared to nurses, they have too much work, we still need more nurses so that we will feel comfortable and work normally.*<br>*It is difficult, despite my condition (few days before giving birth) I'm still working alone."*
			12- No services for prevention of diabetes and HBP	*12- "We rarely organize the awareness campaigns, all we do is, we try to inform patients to be aware of the danger of certain diseases and potential solutions to fight these diseases. So, we do very little in prevention."*
		Meaningfulness (3)	1- Lack of engagement of staff	*1- "The problem is mainly the lack of motivation of the staff we meet in the hospital. It is an internal problem, it is an administrative engagement problem, the staff is not motivated when you start talking about patient empowerment."*
			2- Feeling helpless in how to help patients develop their autonomy to face diseases	*2- "… there is nothing I can do to help them. I at my level, I only take the parameters, I give what I can give to patients. I cannot force the patient to go and do what was said. So, we will only give the advice, it is up to the patient to choose. If he wants healing, he will choose the good road and if he does not want healing, we will give the advice and he will not take seriously, it depends on the patient."*
			3- Lack of enthusiasm for continuous learning on "patient empowerment"	*3- "…I mean that we have weekly rounds that we do a bit continuous training session. But updating knowledge in a very rigorous way is things that require a lot of engagement that it is missing right now. And many of them do not even know how to motivate their patients to become empower."*
	RRG (12)	Internal resources (3)	1- Lack of appropriated skills from some health professionals to empower patients	*1- "The skills of the medical staff, asking a nurse like that to give more of his time to a diabetic or hypertensive patient is a real challenge. This raises a problem of education, even the work of the nurse is in relation with empowerment, in my opinion, we should not wait for that from a nurse for now. We must build this with*

				training tools, communication tools, and perhaps for those who will follow the evaluation tracking tools."
			2- Not enough time for each patient	2- *"We do not have enough time, but for me, we have to create time because we are in front of human beings not animals. So, we have to create time to take care of the entire person."*
			3- No trust between members of the healthcare professional team	3- *"I will surprise you, but sometimes there are a certain people in the staff who do not know how to do it. So, you have to have more confidence in the training that you give to patients than to other member of healthcare professional."*
		External resources (9)	1- Not enough space in the PHCDH to manage all diabetics and hypertensives patients	1- *"... in general the space is too small, yes the location we manage these patients, because the health area of the PHCDH counts about 353160 souls. And it is true that the space it is small, we would like to have a little more space to do a little more, to be well organized."*
			2- Lack of resources for continuing training of health professional	2- *"... we need the resources, because sometimes there are ongoing training sessions that are organized by laboratories, whether in Cameroon, or abroad where the staff can participate, whether it is nurses or doctors, but there is no budget for that."*
			3- Lack of human resources	3- *"... that is what I was saying, it requires resources, so the first thing is human resources, you need staff who can do this job because there's already a lot of activity. Of course, they are involved, they are available, but in terms of human resources to satisfy these patients it is insufficient, but in terms of nurses to satisfy these patients it is insufficient and there is no dietician."*
			4- No dietician at PHCDH	4- *"Any patient with a chronic NCDs should also be referred to a dietician, so at least any hospital had to have a dietician, but we do not have one here. We refer them to a dietician at central hospital, so that they can learn and improve the eating habits but must of the time they do not go there."*
			5- Many products are out of stock	5- *"... they made available many products for the club, and when it finished, we go directly to order more. But lately they were out of stock, the patients of the club were angry, it hurt me because they did not deliver on time, the person who had to come with did not come."*
			6- No storage space for patient's data	6- *"... there is also logistics in terms of the preservation of the material, data. For e.g., sometimes we have patients' files, and it is necessary to keep them well but, we lack space and even when there is space it is not secure. We lack a lot of space and security of all that data in the hospital."*
			7- High cost of products / services	7- *"... we have to try to reduce the costs of care, I think it will help many poor patients and families."*
			8- No budget for the multiplication of existing resources at the hospital to serve the maximum of patients	8- *"... but there are products ready, but not distributed to people because there is no funding to make multiple copy. ... so, the budget constraint makes things difficult. The ideal would have been that every patient has the complete package of all education themes so, if they have problem, they can refer to the document to*

				understand and solve the problem without go to the hospital every time and that is what will help empowerment."
			9- Defective work products / equipment	*9- "... it can happen that the equipment is defective. For e.g., in the service of medicine, you see that we have a lot of hypertensive and diabetics patients, and sometime blood pressure monitor just stop working because it is overused, or it can create bias in dada."*
	Beliefs		/	/
	Satisfaction (5)		1- Waiting time	*1- "The patient's waiting time is too long, because after buying consultation ticket, the patient waits for a long time to see the doctor. He cannot be satisfied; the waiting time must be very reduced for these patients."*
			2- Unstructured patient reception	*2- "The problem with patients is we cannot satisfy them 100% because patients are always scrambling, like I arrived at such time, the doctor did not arrive quickly, I am here since, ... therefore the reception must be taken quickly, the care must also be automatic and then shorten the waiting time for patients."*
			3- The hospital premises are old and dirty	*3- "One of the problems is that the patients are afraid of the hospital, well I remember what was often said, it is not very clean in these hospitals, first thing, they are little afraid here, the hospital is not pretty, the paintings are not well done, the comfort is approximate, the beds are not ..."*
			4- Difficult to navigate in the hospital	*4- "... second thing they get to the hospital they're lost; people do not tell them where to go what to do and now some complain."*
			5- Bad attitude of some nurses	*5- "...they are not soft, they are not polite, so, sometimes patients fear the hospital when they arrive, they are a little afraid there ... and then the behaviour of the staff, you see the patients try to be smiling but they look at them sadly so, there's no welcome there."*
	Adherence and health outcomes (2)		1- No home services, no patient record	*1- "I think if you visited one of these clinics, you found that they did not have home follow-up services and patient record, if there were these services for the older people, the adherence will increase with more people with positive results."*
			2- Moving from public hospital to private	*2- "From a while ago, there was the impression that patients were commercialized, which meant patients are lost to follow up because they go from one point to another, from public to private, they do not always find healing and that is where the contradictions begin."*
**Individual level (25)**	SOC (16)	Intelligibility (4)	1- Low level of education of patients / families	*1- "People are not always educated on patient empowerment, education also plays a role, there are certain things that the patient cannot easily understand if he does not have a certain level of education. If he is not educated it will be very difficult, the educated patient is easy to empower. But we work in areas where patients are mostly non-educated, so, I think we cannot say yet that our patients are empower because when we start from 0, we are not going to get to the top in six months or a year."*

			2- Laziness of patients to understand the disease and take care of themselves	*2- "... we really try to reassure the patients, to explain the disease so that they understand well, but for some of them, whatever we do, they do not understand, they do not want to understand to be adherent."*
			3- Lack of awareness of the existing "diabetes Club" in BHD	*3- "... but I said earlier these projects do not yet have their impact as we receive few hypertensive and diabetic patients here, but we know that population of diabetic hypertensive in Cameroon is growing. So, it shows that the population is not informed. General practitioners, as I said earlier who receives them, do not bring them there, because they do not know the value of sending a patient to the club to fight these diseases."*
			4- Difficulty in learning	*4- "In general learning is difficult, there are some who quickly learn, some who will learn slowly but we insist each time we see them, it is a process we start and restart again ... there are some that are just hard to understand, for some people it is their age, for some it is vascular dementia, etc. these are all things that make it difficult to empower them."*
		Manageability (7)	1- Being in an unfavorable physical environment	*1- "In our area, it is things that are not yet developed in several neighborhoods. There are places, but that are far away, like the "vita course", there are sports clubs, but I think that for the urban environment there is a lot to be done to facilitate elderly people who have problems with low mobility, precisely who also need space to walk."*
			2- Difficulty changing lifestyle	*2- "... the living environment for many, it is my opinion, I do not speak as a doctor but as a person, it is complicated. They cannot adapt diet, it is difficult. What they have more on hand is starch, couscous, to change it is really difficult. Sometime vegetables are expensive, they consume salt a lot when it comes time to reduce, it becomes difficult."*
			3- Living alone	*3- "Many do not manage well because they live alone and those who live far away, the majority actually live alone, so that is the downside. I cannot say that many really live with children or with their family, they are alone, so, follow-up is not good, their diet also is not good, they are trying to eat everything."*
			4- Difficulty using certain devices	*4- "... but at some point, sometimes the manipulation of the devices that are made available to them is not easy because of the eye problems. So, empowering the patient is good but the caregiver must be there to frame if it is possible."*
			5- Attending multiple hospitals at once	*5- "...the patient who move from one hospital to another, or from a traditional medical system to here is a problem, he moves from one health center to another, so, we do not follow him up continuously, it difficult for him to change and sometime his health declines faster."*
			6- Being less involved in the management of the diseases	*6- "... because the specialists are there, because there is the equipment and the technical platform that is needed and because most people or a large number in this city can access the treatment, so, if they do not take their medication, if they do not improve it is probably because they are less well involved."*
			7- Difficulties of being diagnosed and accessing care	*7- "There is need to more awareness and accessibility to diagnosis and care. The patient must have the choice to decide because at present moment the accessibility to care is so difficult that I do not think that the patient even has a choice. Even if*

				he manages to be diagnosed, very often it is accidental. He is diagnosed because he has had a complication and he is obliged to come to the hospital, he had no choice to come."
		Meaningfulness (5)	1- No biopsychosocial care	1- *"... with the participation of nurses, sometimes they feel that their job, what they have to do is administer care and then go back home. So, continuing education in the staff is very important to teach them the psychological aspect, because the patient does not feel that the staff is interested in his whole person."*
			2- Fear of disease	2- *"... for some it is fear, despite everything we do, he cannot be empowered to manage the disease, and this makes them feel powerless even more."*
			3- Losing a family member	3- *"... there are others who lose family members, and this de-empower them even more."*
			4- Not believe in health professionals' competences	4- *"You know it is so hard or difficult when you want to advice patients, because they think you are going to slowly killing them. They think you do not know what you are talking about. If there were things to improve, that would be, have the trust of patients.*
			5- Unwillingness to change lifestyle to regain health	5- *"Unwillingness, because if a patient is told to stop smoking or alcohol or what other cardiovascular risk factor, if he does not have the will, he will not do so, even if he has the psychological and family financial support to do so. Others refuse outright and say they do not want to be addicted to drugs, they quit as they please."*
	GRR (5)	Internal resources (3)	1- Difficulty changing / keeping healthy lifestyle	1- *"... about the diet, others say they do not eat well because they do not see, that is why they have no choose to eat everything. So, we cannot force them to respect the diet, we cannot because we are not with them most of the time. They do not respect the diet because they have many troubles (laughs) so that is it, they say it is difficult."*
			2- Ageing	2- *"... they cannot walk, they're already at a certain advanced age, are you going to ask a mom to get up to go for a walk, our moms these days know that it is only the field that kept them active, that is why I'm telling you it is difficult. They will ask you, my daughter at my age I am still going to walk? or play sports? No."*
			3- Lack of determination / laziness	3- *"It is complicated, there are those who follow what you say and others when you ask not to eat this, they will not. Especially you know these people love to eat, they will tell you that I was hungry, I could no longer bear that is why I ate. So, when you find that the patient is stubborn, from time to time you speak even if you find that he does not follow the advice."*
		External resources (2)	1- Lack of help/support or being solely responsible for his disease	1- *"Yes autonomy is not only about patient but there is also the family so, more often if the family is involved, the results are better. I always like to talk in the presence of closer family member, the one who is often with the patient. ... because that is the key factor. As today I received a case where the patient is not empowered, not controlled because he lacks support from his family, it is the first disability."*

			2- Lack of financial to purchase recommended medicines / healthy foods	*2- "The patients still pay for the drugs and services out of their own pockets. The categories of patients who come to the hospital are different, there are few people who have private health assurance and many people who do not have. So, they must pay for their care and products. Many of them are poor, the most limited factor, because there is follow-up and appointments, also planning exams to do, diet, but sometimes they have no money."*
	Beliefs (3)		1- Believing that the disease is caused by spiritual forces	*1- "... explain the diseases to them, because patients sometimes they expect miracles, I explain to them what we do, it is not spiritual, it is scientific the causes of the disease."*
			2- Believing that traditional medicine is the solution	*2- "... it is her husband, they say we are going to treat her with traditional medicine, but we can see she is not controlled at all, and there are already complications."*
			3- Believing that there is no treatment, that it is an incurable disease	*3- "... so to empower patients, we have to see how people live in society, in the African context whatever we do, they will always think that we cannot cure this disease, they think that it is a disease for life."*
	Satisfaction		/	/
	Adherence and health outcomes (1)		1- Difficult to came back for follow-up appointment	*1- "... most of our patients are patients are retired, e.g., patients who stop their treatment for a year or two years and then they come back with complications, it difficult to follow up those who come with complications."*
