# Supplementary material for: Healthcare providers’ and policymakers’ experiences and perspectives on barriers and facilitators to chronic disease self-management for people living with hypertension and diabetes in Cameroon
Source: BMC Prim Care. 2022 Nov 21;23:291. doi: 10.1186/s12875-022-01892-8 (PMC9680136; doi:10.1186/s12875-022-01892-8)
Supplement: Supplementary file 4 — Additional file 4. Facilitators to patient empowerment in the management of their diabetes/hypertension. [file 12875_2022_1892_MOESM4_ESM.pdf]

Additional file 4. Facilitators to patient empowerment in the management of their diabetes/hypertension

Health system	Mega codes (8)	Sub-codes (15)	Themes (42)	Representative quotes
**Central level (8)**	SOC (7)	Intelligibility (2)	1- Organization of seminars / workshops for continuing training of health professionals	*1- "... talking about the Ministry of Health, from time to time he runs small seminars that we can talk about diabetes and HBP, these lessons help to make sure that we are up to date, and with the doctors in the hospital, from time to time they do the retraining for nurses."*
			2- Organization of free awareness and screening campaigns for population	*2- "We often organize screening campaigns in the community, which allows the new case detected to be directed to the health centers. Without mass awareness, screenings people are often not aware of their diseases problem, this helps to highlight new cases, which are often people who do not complain about anything and arrives at the hospital with complications."*
		Manageability (5)	1- Partnership with NGOs expert in the management of diabetes and HBP	*1- "... OK we have a technical and financial partner that supports the delegation. It is involved in the training of staff, equipment, and the education of the patients. This project is in line with the guidelines of the Ministry of Health, it is a collaboration agreement with several NGO experts in the management of NCDs."*
			2- Introduction of PEC clubs in several hospitals including PHCDH, initiated by an NGO	*2- "... so the diabetic patients club is there and allows to coordinate these patients and archiving of information that concerns these patients, because in everything we do, we have to have the information to see in which direction things go, see if it works or not. So, the club is not the initiative of the hospital, but the club is a more NGO initiative."*
			3- Development of HTN and T2D management protocols for nurses (low-resource settings)	*3- "We developed protocols for the management of hypertension and diabetes, ... I had the privilege of leading the development of protocols and so, we wrote protocols for diabetes, hypertension and other chronic diseases at the district hospital level for nurses."*
			4- Direct funding of hospitals by the Ministry of Finance under the supervision of the Ministry of Health	*4- "... the Ministry of Finance, under the supervision of the Minister of Health send the budget directly to the hospital, but it is the Minister of Health who submitted their request. But it is the hospital who asked for that money. So, the hospital must start the activity and submit to the minister for validation, because it is the minister who assumes it."*
			5- Implementing strategies to reduce drug costs for patients	*5- "Since we realized that drugs were very expensive for patients, we arranged to get the drugs from the wholesalers and put them in the hospital pharmacies, because this would remove some intermediate fees that pharmacists take and make it cheaper for patients."*
		Meaningfulness	/	/
	GRR (1)	Internal resources	/	/

		External resources (1)	1- Use partnerships to subsidize certain products and services	*1- "... with the support of the "camboj project" which is led by Professor X, our hospital has been equipped with some good technology to the management of these patients, which ensures that the inputs in diabetes control are available, and the patient will pay nothing. ... until then what we appreciated are the prices of the insulin, before it was at 3000f to 5000f for texting blood sugar, now it is at 500f, I think we succeed the challenge."*
	Beliefs	/	/	/
	Satisfaction	/	/	/
	Adherence and health outcomes	/	/	/
**Organizational level (19)**	SOC (12)	Intelligibility (7)	1- Better understanding of "patient empowerment"	*1- "As a specialist, it is known that the management of chronic diseases depends in large part on the patient's ability to understand the problem himself and manage, understand his diagnosis, objectives and understand his follow-up. The interest for him is to take his medication and follow-up with doctor recommendations to therefore become "EXPERT" of his pathology. This is overall how we perceive self-management of chronic diseases here."*
			2- Self-training of health professionals	*2- "... in my personal studies, I was able to revisit it, especially in diabetic patients because, follow-up is more important, there are problems of manipulation of insulin on those who are on treatment for self-medication, diet also, physical activities, advice for weight loss, so, what I read I learned on the internet. ... yes, we continue to look for the different tricks, we look for different formations. So, we're always looking more and more for how to improve our patient's health, make them even more empower so we are always on reflection to improve."*
			3- Being polyglot	*3- "For those who speak French it OK, and I can say that for me I have no problem, I am polyglot so, I speak several languages, sometimes I use the vernacular languages to communicate the message. I can also use the patient's language for their educational needs."*
			4- Presence of "diabetic club" for education of diabetes and hypertensive patients	*4- "For diabetic and hypertensive patients, there is a club where patients are educated, they are taught how to live with the disease, how to manage it. That is the role of the club, even before we start taking their parameters, to control their blood sugar we first educate them, we explain to them in relation to their illness, the consequences if they do not respect the diet, medications and all recommendations, we always explain that to them."*
			5- Distribute patient education pamphlets	*5- "... there is for example the diet, sometimes we distribute the pamphlets to them, we distribute, we give everyone how to eat pamphlet, so, the person tries to eat that way. ... we provide a sheet with a general diet, what foods they should avoid or should eat."*

			6- Importance of empowering the family's patient too	*6- "Yes, I say that with the patient's consent we see his family, we also educate his family about the pathology, because it is not only the patient who will make the food at home, the family must know the quality of food to do to give the patient, that is why they must be empower too, they must be detected also to see if they are not sick too, because sometime the disease is hereditary."*
			7- Continuing training for nurses/paramedics on prevention and management of diabetes/hypertension	*7- "Therapeutic education, it is true I do continue education for nurses, on how the patient should eat, the lifestyle with the disease, so in everything we do, we try to involve them. I recently had to train health workers who come from different backgrounds and fortunately I starts with our nurses here. What we also do every day is educate them during the consultation rounds."*
		Manageability (2)	1- Tasks shifting between doctors and nurses	*1- "In general we do the "task shifting", we empower patients but also staff. Of course, those who come do not have that spirit, but in a short time they get involved in everything we do, we explain to them the merits of everything we are doing and the logic. So, they develop the capabilities, sometimes they take certain initiatives, of course when it is done well, we encourage them, when it is not well done, they are encouraged to do well and teaches them without harming. To do therapeutic education, for e.g., nurses must also participate on how the patient must eat, lifestyle with the disease, so in everything we do we try to involve them."*
			2- Development of emergency management protocol for nurses	*2- "The staff works 24 hours a day, there are the permanent staff, even if a person arrives in the night, there is a protocol in the service that are displayed on the wall on the management of cases, if even the patient comes from elsewhere where he has not been follow up well, we take the protocol, we follow, and in the morning we present the case to the Doctor to evaluate what we have done if we need to improve."*
		Meaningfulness (3)	1- Build a relationship of trust between health professionals and patients	*1- "A good relationship, yes they have a very good contact with doctors and the doctors also are welcoming and humble. They listen to them because that is the good thing to do, listen to the patient. You have to listen to him, if you listen carefully to your patient, you will gain his trust, and this trust we have it here between our doctors and patients."*
			2- The ethical values of the medical profession	*2- "It is said that our profession is a priestly profession so, we are at the service of the other we must find time for them. So, in terms of consultations, education, risk factors, prevention, training of staff, we must be able to find time because it is first for the patient and not for us."*
			3- Moral assistance to patients	*3- "...we play a kind of second role and assist them morally specially for those who cannot pay for their care, we assist them, and this motivates them a lot to follow the doctor plan."*
	GRR (4)	Internal resources (2)	1- Sharing knowledge / expertise between health professionals	*1- "I do it every day with medical students and young colleagues when they are here, when we have a case, I give them my point of view how they should approach the patient and so on, to have total success in the management by helping the patient who is going to be aware, believes positive, I share this with colleagues."*

			2- Having experience with patients with chronic NCDs	2- *"... it is much more my personal experience with these patients because I spent a lot of time with them, their daily experience. I think with that there will be success especially in the African environment, so there is not really a guideline in relation to that, it is just experience."*
		External resources (2)	1- Give products (drugs) to patients who cannot afford	1- *"... we help them, especially patients who cannot afford products, we give them appropriate treatment and sometimes we help them with the drugs, we give them the drugs for free to really help them in their care management."*
			2- Availability of work equipment / a good technical platform	2- *"... we are lucky that we were provided with equipment to work: ... so in terms of equipment ... there's the scale to gain the patient's weight."*
	Beliefs		/	/
	Satisfaction (3)		1- Good welcome, care, and follow-up of patient	1- *"When they feel healthy, they feel good, they are happy to see that we have being taken care of them properly by actually explaining what it is and why they have these chronic pathologies so, to my knowledge I think there is satisfaction. ... For the number of patients, we have followed in recent years, they are mostly satisfied. I believe the happiness and satisfaction of patients have a lot to do with the quality of patient's welcome and follow-up."*
			2- Progress in diabetes and HBP management	2- *"I think things have changed a lot, that is, ten years ago, when I was in the central hospital even when I was here, ... now there are already the devices, we have a whole budget to take care of patients who suffer from diabetes and hypertension, so really things have much improvement and patients are satisfied."*
			3- Dynamism / energy of diabetes club	3- *"The follow-up at the hospital helps them because we organized a diabetic and hypertensive club for these people, it is every Thursday, they come to the appointment, I explain things to them and you see it changes the atmosphere, there are a lot of them who like the good vibes. When they arrive there are prayers, songs for animation and love. So, when they are at home, they say Thursday is what day, I must get ready to go to the club, so it really helps them."*
	Adherence and health outcomes		/	/
**Individual level (15)**	SOC (8)	Intelligibility (3)	1- Detailed explanations from health professionals	1- *"The process that works is the explanation, because it is difficult to tell the patient that he is going to be dependent on drugs all his life, go very gently until the patient actually understands his disease. We go into pathophysiology so that they understand what it is a damage to the vessels, the pathophysiology helps them to really understands what it is."*
			2- A better understanding of the disease and its complications	2- *"Once the patient understands the need, he understands the disease and the risks of complications, he starts self-manage the diseases, things become very easy for him and it also makes life easy for us. Then the success is important even if it is not total, it is encouraging."*

			3- Advice between patients and knowing that they are not the only ones to have this disease	*3- "They do that, they give advice to other patients, it is between patients, because the staff asks someone who has something to share like about complications on the feet diabetes? Then, a patient can get up, explain us and we correct only, the other patients now know that this pathology there, it is not just me who has, there's a lot of people out there who have that."*
		Manageability (2)	1- Having a notebook and writing down everything they eat	*1- "... for the diet we try to ask, I ask them to write down in a notebook what they eat, and we try to see what the proportions are, so knowing a little well the lifestyle of the patient we get to get some results especially with the younger."*
			2- Have a biopsychosocial approach in management of the diseases	*2- "When I receive the patient, I say hello, we call him by his name, ask him some personal questions about life, work, friends, how it goes and everything. Now we introduce why he is here, then we examine him. Patients like it, he feels that we take care of him, we are interested in what he has, so listen to him and then explain to him a little, because patients sometimes they expect miracles. We explain to him a little what we do it is not witchcraft, it is the process; why we will take this or that test in cases of diabetes and high blood pressure, ... and then patients like when we give them non-pharmacological treatment measures."*
		Meaningfulness (3)	1- Fear that the disease will get worse or die	*1- "... the evolution of the disease, when people start to have more complications, they take things more seriously, so the progression of the disease also contributes at that time to the empowerment of patients, he starts to take things seriously to be able to self-manage the disease."*
			2- Accepting the disease	*2- "When the patient accepts the disease, that the disease is a chronic disease, he participates because you can tell him what to do. When he accepts that now I must eat this and not this, I must walk, that means yes, he begins to be empowered, and follows his treatment." ...*
			3- Wanting to live longer	*3- "I told you, others sometimes want to get discouraged but because he wants to live longer, we tell them you cannot live longer if you do not follow treatment plan. We motivated them to act well to respect what they are asked to do to heal."*
	GRR (5)	Internal resources (2)	1- Need to check out his health	*1- "Every Thursday they are here for control, they tell themselves that at a certain age, because they see others who have died in their family because of diabetes, automatically someone who comes from the family will say that I am already a certain age, I have not been declared diabetic or hypertensive but, it is the better I go to do my check out. It is not just diabetics who are at the club, there are older people who come regularly, due to risk factors."*
			2- Being responsible of the diseases	*2- "When a patient realizes that I am able to see if my blood pressure is high or low or normal, here he is in power, he really has a capacity building and he can make a decision, he can call his doctor to tell him that my blood pressure has gone up or my blood pressure has gone down too much. With these elements, they better appropriate the elements that allow them to improve their condition when they are sick."*
		External resources (3)	1- Being married / have a close family members or community support	*1- "We also found for some patients that, we could not use them when talking about empowerment, we had to go through the family. For example, wives do it*

				very well for their husbands, sometimes it is the children and it allows to manage everything around the patient."
			2- Have their own equipment to measure blood glucose / pressure and medications	*2- "When it comes to diabetes, he is encouraged to have his own device, so he can monitor things and make him independent. Like have his own drugs will help manage his diabetes, and he can then control the diseases, and knows that here are the things to do every time."*
			3- Reducing the prices of products and services	*3- "Indirectly, the low cost of inputs, the patient becomes very autonomous in the sense that these things are there those who do not have the device easily at any time they come, he gives 500f, we do his blood sugar quietly and here he is encouraged to participate in his care."*
	Satisfaction		/	/
	Belief		/	/
	Adherence and health outcomes (2)		1-Attitude of health professionals with patients	*1- "... I can say that two things are very important: there is attitude towards the patient, you have to give him confidence, he has to feel listen, he has to feel that there is hope, that he can heal, that hypertension and diabetes are not the end of the world, it is common and then that he will have a good follow up, he will find solutions."*
			2- Evolution of the disease	*2- "... because the treatment evolves, when the patient comes at his appointment, you will see that he feels good, that he is doing well yes, the factor favoring, what I saw is that when the patient takes his drugs well, the prescription well, he does not skip it, he will be good."*
